# Crosstalk between the Circadian Clock and Innate Immunity in Arabidopsis

**DOI:** 10.1371/journal.ppat.1003370

**Published:** 2013-06-06

**Authors:** Chong Zhang, Qiguang Xie, Ryan G. Anderson, Gina Ng, Nicholas C. Seitz, Thomas Peterson, C. Robertson McClung, John M. McDowell, Dongdong Kong, June M. Kwak, Hua Lu

**Affiliations:** 1 Department of Biological Sciences, University of Maryland Baltimore County, Baltimore, Maryland, United States of America; 2 Department of Biological Sciences, Dartmouth College, Hanover, New Hampshire, United States of America; 3 Department of Plant Pathology, Physiology, and Weed Science, Virginia Tech, Blacksburg, Virginia, United States of America; 4 Department of Cell Biology and Molecular Genetics, University of Maryland, College Park, Maryland, United States of America; 5 Department of Cell Biology and Molecular Genetics, Department of Plant Science and Landscape Architecture, University of Maryland, College Park, Maryland, United States of America; 6 Department of Plant Molecular Systems Biotechnology and Crop Biotech Institute, Kyung Hee University, Yongin, Republic of Korea; Massachusetts General Hospital, Harvard Medical School, United States of America

## Abstract

The circadian clock integrates temporal information with environmental cues in regulating plant development and physiology. Recently, the circadian clock has been shown to affect plant responses to biotic cues. To further examine this role of the circadian clock, we tested disease resistance in mutants disrupted in *CCA1* and *LHY*, which act synergistically to regulate clock activity. We found that *cca1* and *lhy* mutants also synergistically affect basal and resistance gene-mediated defense against *Pseudomonas syringae* and *Hyaloperonospora arabidopsidis*. Disrupting the circadian clock caused by overexpression of *CCA1* or *LHY* also resulted in severe susceptibility to *P. syringae*. We identified a downstream target of CCA1 and LHY, *GRP7*, a key constituent of a slave oscillator regulated by the circadian clock and previously shown to influence plant defense and stomatal activity. We show that the defense role of CCA1 and LHY against *P. syringae* is at least partially through circadian control of stomatal aperture but is independent of defense mediated by salicylic acid. Furthermore, we found defense activation by *P. syringae* infection and treatment with the elicitor flg22 can feedback-regulate clock activity. Together this data strongly supports a direct role of the circadian clock in defense control and reveal for the first time crosstalk between the circadian clock and plant innate immunity.

## Author Summary

Plants are frequently challenged by various pathogens. The circadian clock, which is the internal time measuring machinery, has been implicated in regulating plant responses to biotic cues. To better understand the role of the circadian clock in defense control, we tested disease resistance with Arabidopsis mutants disrupted in *CCA1* and *LHY*, two key components of the circadian clock. We found that consistent with their contributions to the circadian clock, *cca1* and *lhy* mutants synergistically affect resistance to both bacterial and oomycete pathogens. Disrupting the circadian clock caused by overexpression of *CCA1* or *LHY* also results in severe disease susceptibility. Thus, our data further demonstrate a direct role of the circadian clock mediated by CCA1 and LHY in defense regulation. We also found that CCA1 and LHY act independently of salicylic acid mediated defense but at least through the downstream target gene *GRP7* to regulate both stomata-dependent and -independent pathways. We further show that defense activation by bacterial infection and the treatment with the elicitor flg22 can also feed back to regulate clock activity. Together our study reveals for the first time reciprocal regulation of the circadian clock and plant innate immunity, significantly expanding our view of complex gene networks regulating plant defense responses and development.

## Introduction

Plants are challenged by various pathogens on a daily basis. Accumulating evidence implicates a role of the circadian clock in regulating plant innate immunity. The circadian clock is the internal time measuring machinery important for plant growth and development. However, our understanding of the molecular basis of how the circadian clock controls plant innate immunity is still in its infancy.

Plants have evolved various mechanisms, some pre-formed and others induced, to ward off pathogen invasion. An example of pre-formed surface structures is the stomate, the natural opening important for photosynthetic gas exchange. This opening can provide a portal for pathogens to enter leaves; however, plants can also control the aperture of stomata to physically limit pathogens [1], [2]. One type of induced defense is activated when plants recognize pathogen-associated molecular patterns (PAMPs), which are conserved molecules or structures present in groups of related microbes. This defense, also termed PAMP-triggered immunity (PTI), can be highly effective against non-adapted pathogens and provides a basal level of defense even against adapted pathogens [3], [4]. Another type of induced defense is activated by plant resistance (R) proteins, which specifically recognize secreted pathogen effectors and subsequently activate effector-triggered immunity (ETI). ETI, also termed *R* gene-mediated resistance, is a stronger and faster elaboration of PTI, and frequently results in hypersensitive cell death at the infection site [5], [6], [7]. The small molecule salicylic acid (SA) has been linked to signal transduction in PTI and ETI [8], [9], [10].

The circadian clock has profound influence on the fitness of organisms [11], [12], [13], [14], [15], [16]. The core of the circadian clock is the central oscillator, which in Arabidopsis, is composed of multiple interconnected negative feedback loops that orchestrate biological adjustments independently of external stimuli [17], [18]. Of these clock components, CIRCADIAN CLOCK ASSOCIATED1 (CCA1) and its close homolog LATE ELONGATED HYPOCOTYL (LHY) are transcription factors that are involved in multiple feedback loops and function synergistically to regulate clock activity [19], [20], [21].

The role of the circadian clock in controlling plant innate immunity has long been proposed based on circadian-regulation of defense gene expression [22], [23], [24], [25], [26]. Direct evidence from several research groups has recently emerged to support such a role of the circadian clock. Under free running conditions, wild type Arabidopsis exhibits temporal oscillations in susceptibility to *Pseudomonas syringae* infection, which are disrupted by overexpression of *CCA1* [27]. Misexpression of several clock genes, including *CCA1*, compromises resistance to the bacterial pathogen *Pseudomonas syringae* and/or to the oomycete pathogen *Hyaloperonospora arabidopsidis* (*Hpa*) [27], [28], [29]. Interestingly, although *lhy* mutants exhibit similarly shortened circadian period as *cca1* mutants, LHY was not shown to play a defense role against *Hpa* [28]. This raises the question of whether CCA1 is a dual function protein, affecting both the circadian clock and other non-clock related processes, as shown in the case of another central oscillator component GIGANTEA [30]. *cca1*-conferred disease susceptibility might be attributed to a role of CCA1 in regulating non-clock related processes rather than to its direct involvement in the circadian clock [31].

To better understand the role of CCA1 and LHY-mediated circadian clock in defense control, we tested plants misexpressing *CCA1* and/or *LHY* for disease resistance to *P. syringae* and *Hpa*. We show that *CCA1* and *LHY* loss-of-function mutants synergistically affect basal resistance and *R* gene-mediated defense against both pathogens. Disrupting the circadian clock caused by overexpression of *CCA1* or *LHY* also results in severe disease susceptibility to *P. syringae*. The defense role of CCA1 and LHY against *P. syringae* is at least partially through circadian control of stomatal aperture but is SA-independent. Furthermore, we found that clock activity is modulated by *P. syringae* infection or treatment with the elicitor flg22. These data further establish the role of the circadian clock in defense control and for the first time reveal crosstalk between the circadian clock and plant innate immunity.

## Results

### The effect of CCA1 and LHY on clock activity can manifest in LL and LD

**Figure 1:**
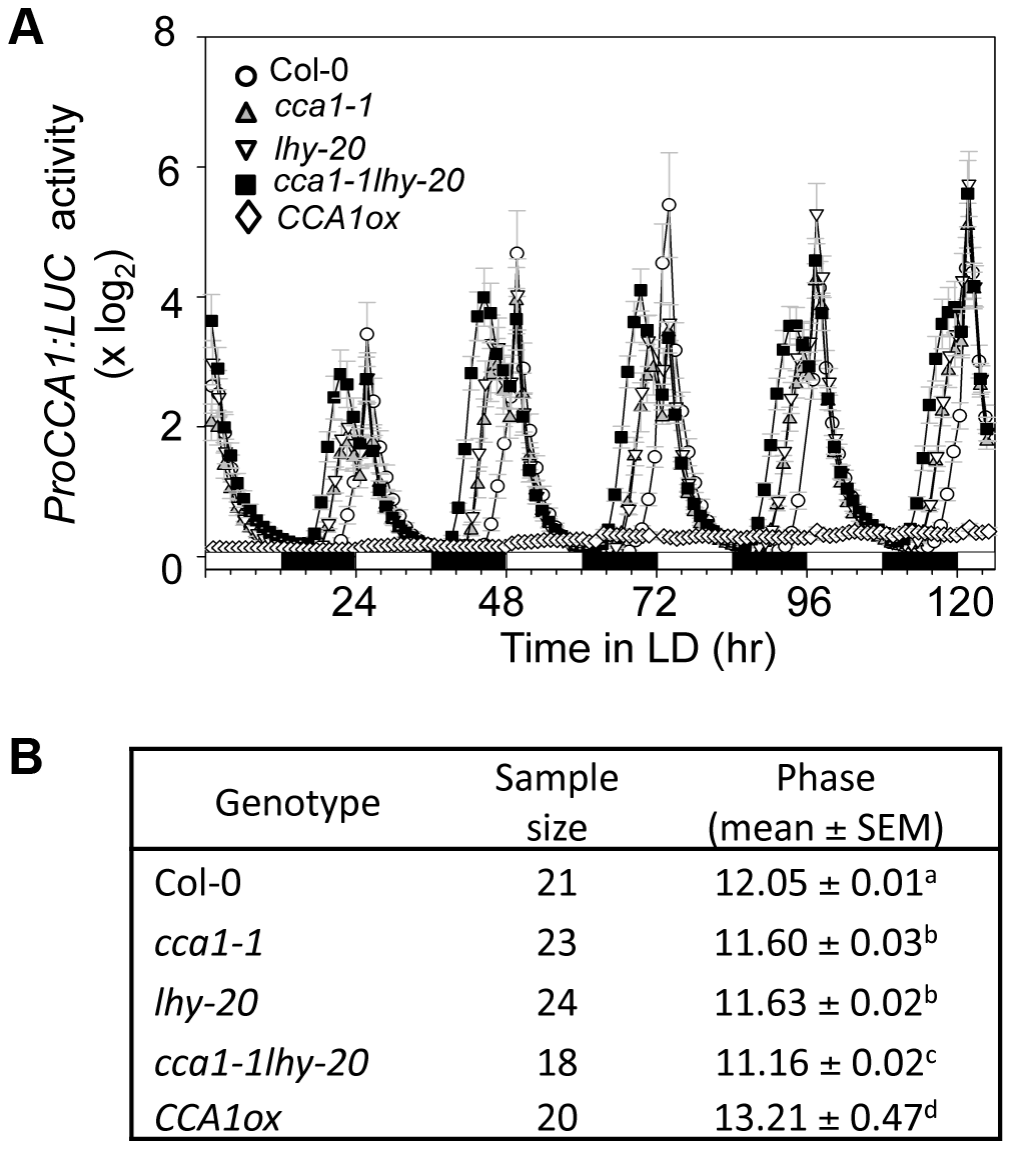
Clock activity of plants misexpressing *CCA1* and/or *LHY* is disrupted in LD. Eight-day-old seedlings of Col-0, *cca1-1*, *lhy-20*, *cca1-1lhy-20*, and *CCA1ox* expressing *ProCCA1:LUC* reporter were grown from germination in 12 hr light/12 hr dark cycles at 22°C. Luciferase activity was recorded with a Packard TopCount luminometer in LD at 22°C. (**A**) Mean circadian traces for *ProCCA1:LUC* activity. (**B**) Summary of phase value for *ProCCA1:LUC* in each genotype. Standard error of the mean (SEM) (n = 12–24) was used for (**A**) and (**B**). Letters indicate significant difference among the samples (P<0.05; Student's t-test).

To evaluate defense roles of CCA1 and LHY, we constructed the *cca1-1lhy-20* mutant via a genetic cross in a Col-0 background that also contains the *LUCIFERASE* reporter gene driven by the *CCA1* promoter (*ProCCA1:LUC*). The single loss of function mutants, *cca1-1* and *lhy-20*, have shortened circadian periods of *ProCCA1:LUC* expression in constant light (LL) [11]. In LL, we confirmed that *cca1-1lhy-20* had a much-shortened period (19.9±0.11 hr), compared with wild type (wt) Col-0 (24.4±0.09 hr) (Figure S1A and [19]). Although experiments in LL are important for establishing the involvement of the circadian clock in specific phenotypes, such experimental conditions can also be limiting. In entraining conditions (e.g., a 12 hr L/12 hr D cycle; LD), the altered period of clock mutants like *cca1-1* and *lhy-20* is not seen due to the entraining cycle, which imposes a 24 hr period (Figure 1). The clock remains important in such LD conditions, though, because the clock determines the phase of specific events with respect to as dawn and dusk. Mutants with altered period in LL typically exhibit altered phase in LD, with short period mutants exhibiting a leading (early) phase and long period mutants exhibiting a lagging (late) phase [32]. Moreover, interactions between the endogenous circadian clock and external LD cycles can results in phase differences, sometimes dramatic, when measured in LD versus LL. For example, the phase of maximal hypocotyl elongation during early seedling growth was shifted 8–12 hours between LD and LL conditions [33], [34]. In their natural environment, plants do not usually encounter LL. Therefore in evaluating the role of the circadian clock on plant defense against pathogens, it is critically important to study plant-pathogen interactions in LD and to consider the potential influence of the circadian clock on the phases of rhythmic events that might influence the plant response to pathogen challenge.

We show here that in LD the phases of *cca1-1* and *lhy-20* single mutants were leading with respect to that of wild type Col-0, and that the *cca1-1 lhy-20* double mutant exhibited a much earlier phase than either single mutant, consistent with the synergistic contribution of CCA1 and LHY in regulating clock activity (Figure 1 and Figure S1B). Early phase was also reported with other *cca1lhy* mutants [20], [21]. In addition, we found that plants overexpressing *CCA1* (*CCA1ox*), which display arrhythmic clock activity in LL [35], also showed arrhythmic expression of *ProCCA1:LUC* in LD with an acute peak in response to lights on (Figure 1 and S1B). Low *ProCCA1:LUC* activity in *CCA1ox* is consistent with CCA1 being a negative regulator of its own expression [35]. These results emphasize that altered function of the circadian clock can manifest in both LL and LD conditions.

### CCA1 and LHY contribute synergistically to resistance to *P. syringae*

**Figure 2:**
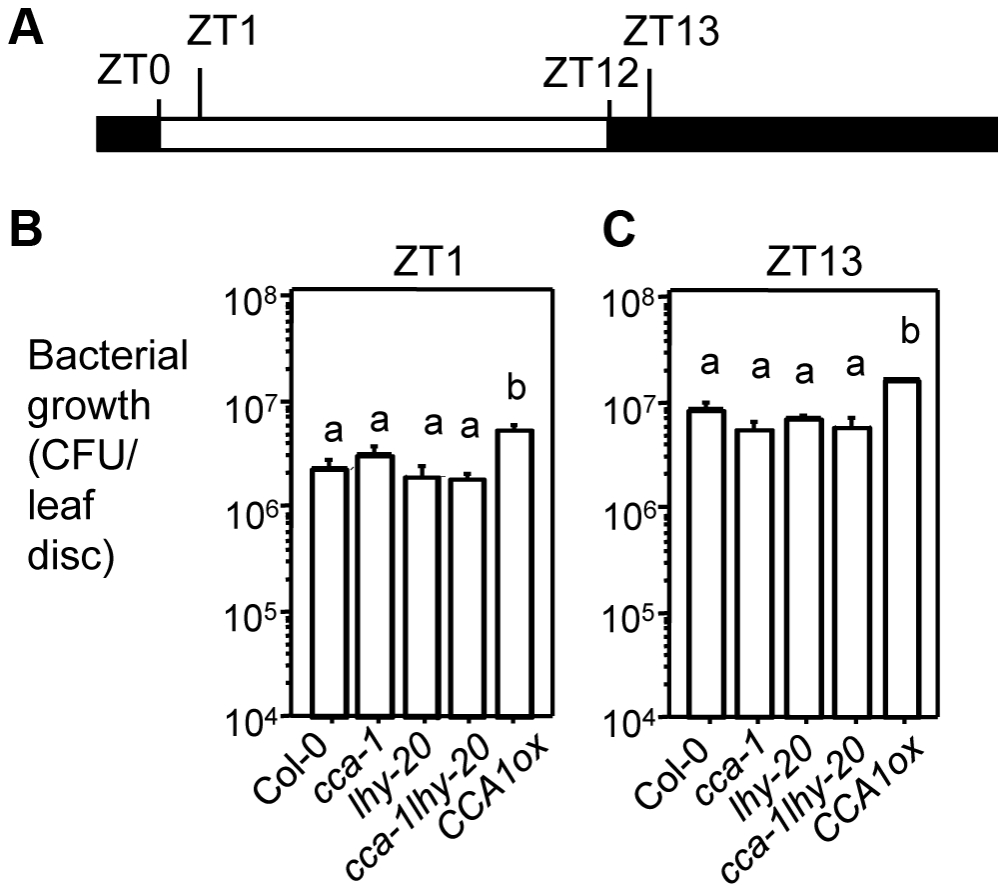
Bacterial growth in plants infiltrated with *Pseudomonas syringae pv.* *maculicola* strain DG3 (*PmaDG3*). (**A**) Time scheme used in this report. The white box indicates the light period and black boxes indicate dark periods. (**B**) ZT1 infection. (**C**) ZT13 infection. In 12 hr L/12 hr D (LD), 25-day-old plants were grown and infected by infiltration with *PmaDG3* at 1×10^5^ colony forming unit (CFU)/ml. Bacterial growth was assessed at 3 dpi. Data represent the average of bacterial numbers in six samples ± standard error. Log transformed bacterial growth was used in statistical analysis (Student's t-test). Letters indicate significant difference among the samples (P<0.05). These experiments were repeated three times with similar results.

To test disease resistance of *cca1-1* and *lhy-20* plants, we performed infection experiments at Zeitgeber Time 1 (Zeitgeber Time is the time relative to dawn; ZT1 is 1 hr after lights on) or ZT13 (1 hr after lights off), two times of day associated with drastic changes of light regime. Plant leaves were pressure-infiltrated with virulent *P. syringae* pv. *maculicola* ES4326 strain DG3 (*PmaDG3*) [36]. The infected plants were placed in either LD or LL. Bacterial growth assays at 3 days post infection (3 dpi) revealed no significant difference among Col-0, *cca1-1*, *lhy-20*, and *cca1-1lhy-20* in either LD or LL (Figure 2 and Figure S2).

**Figure 3:**
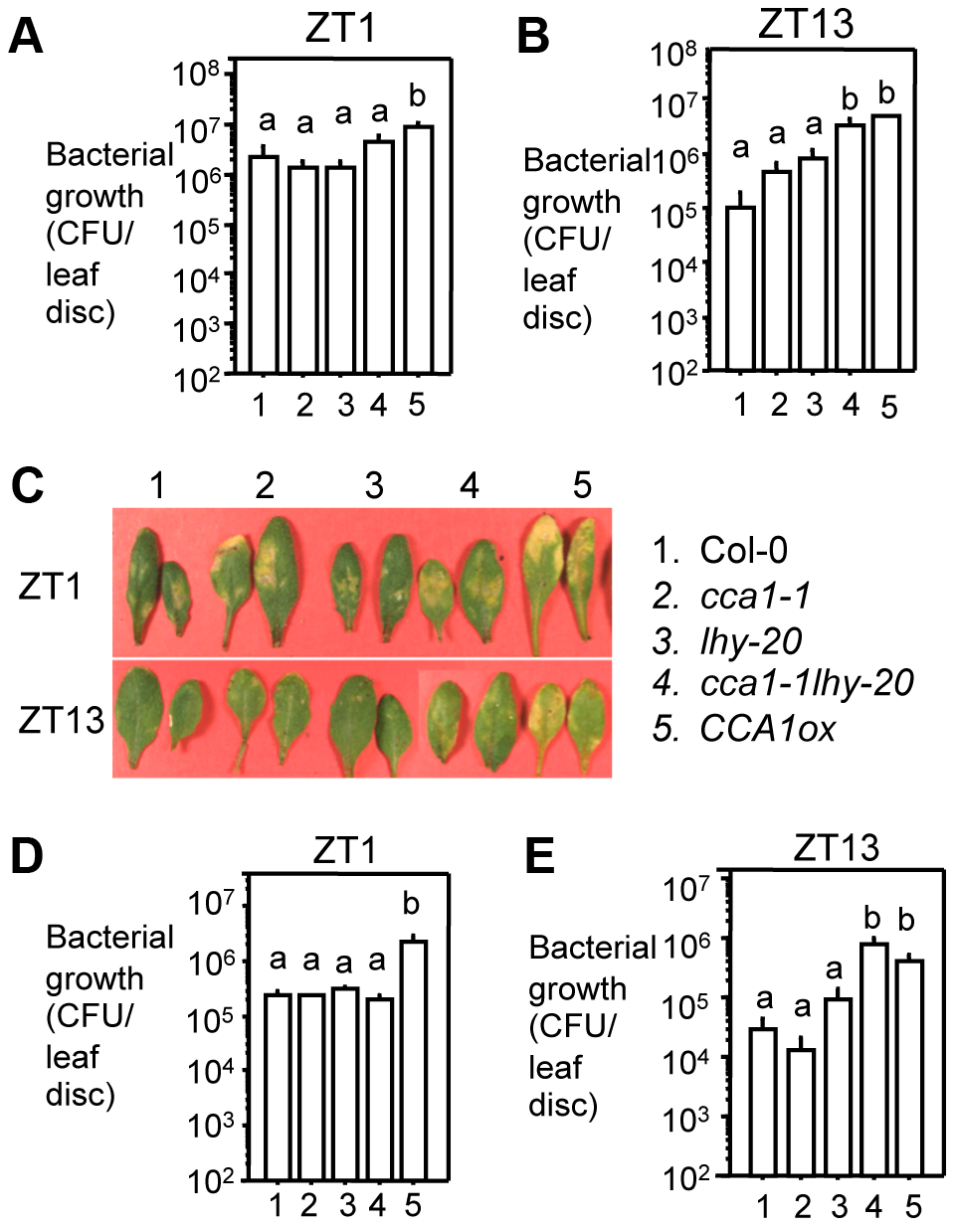
Bacterial growth in plants spray-infected with *P.* *syringae*. (**A**) ZT1 infection with *PmaDG3.* (**B**) ZT13 infection with *PmaDG3.* (**C**) Pictures of infected leaves from (**A**) and (**B**) at 4 dpi. (**D**) ZT1 infection with *PmaDG6.* (**E**) ZT13 infection with *PmaDG6.* Twenty five-day-old plants were infected by spraying with the virulent strain *PmaDG3* or the avirulent strain *PmaDG6* (1×10^8^ CFU/ml) at ZT1 or ZT13. Bacterial growth was assessed at 3 dpi. Data represent the mean bacterial numbers ± SEM (n = 6). Letters indicate significant difference among the samples (P<0.05; Student's t-test). These experiments were repeated three times with similar results.

Under natural conditions, *P. syringae* enters the apoplast of leaves through openings such as stomata and wounds. It is known that stomatal aperture is regulated by the circadian clock [37], [38]. Therefore, infiltration of bacteria directly into plant tissue might bypass the influence of the circadian clock on stomatal defense. To test this possibility, we spray-infected with *PmaDG3* Col-0, *cca1-1*, *lhy-20*, and *cca1-1lhy-20* at ZT1 and ZT13 in LD. We found that Col-0 supported over 10-fold more bacterial growth with ZT1 infection than with ZT13 infection (Figure 3A and 3B), suggesting that Col-0 is more resistant at night than at dawn when spray-infected. Although we did not observe significant difference in bacterial growth between Col-0 and *cca1-1* and *lhy-20* single mutants, the double mutant *cca1-1lhy-20* showed enhanced susceptibility to *PmaDG3* when sprayed at ZT13 (Figure 3A to 3C). Consistent with this result, we found that *PmaDG6* (an avirulent strain recognized by the resistance protein RPS2 in Col-0) [36]) grew significantly more in *cca1-1lhy-20* than in Col-0 and the single mutants with ZT13 infection (Figure 3D and 3E). Together these data suggest that CCA1 and LHY share redundant functions to regulate both basal and RPS2-mediated defense against *P. syringae*.

### Overexpression of *CCA1* or *LHY* confers enhanced susceptibility to *P. syringae*

To further substantiate the role of CCA1 and LHY in defense regulation, we tested disease resistance of plants overexpressing *CCA1* (*CCA1ox*) or *LHY* (*LHYox*), which were shown to have arrhythmic clock activity in LL [35], [39]. *CCA1ox* plants also exhibited clock arrhythmicity in LD (Figure 1 and S1B). Disease resistance assays indicate that *CCA1ox* plants were more susceptible to *PmaDG3* than Col-0 with infiltration infection in LD or LL (Figure 2 and S2). *CCA1ox* plants were also more susceptible than Col-0 to *PmaDG3* and to *PmaDG6* when spray-infected at ZT1 or ZT13 in LD (Figure 3).

**Figure 4:**
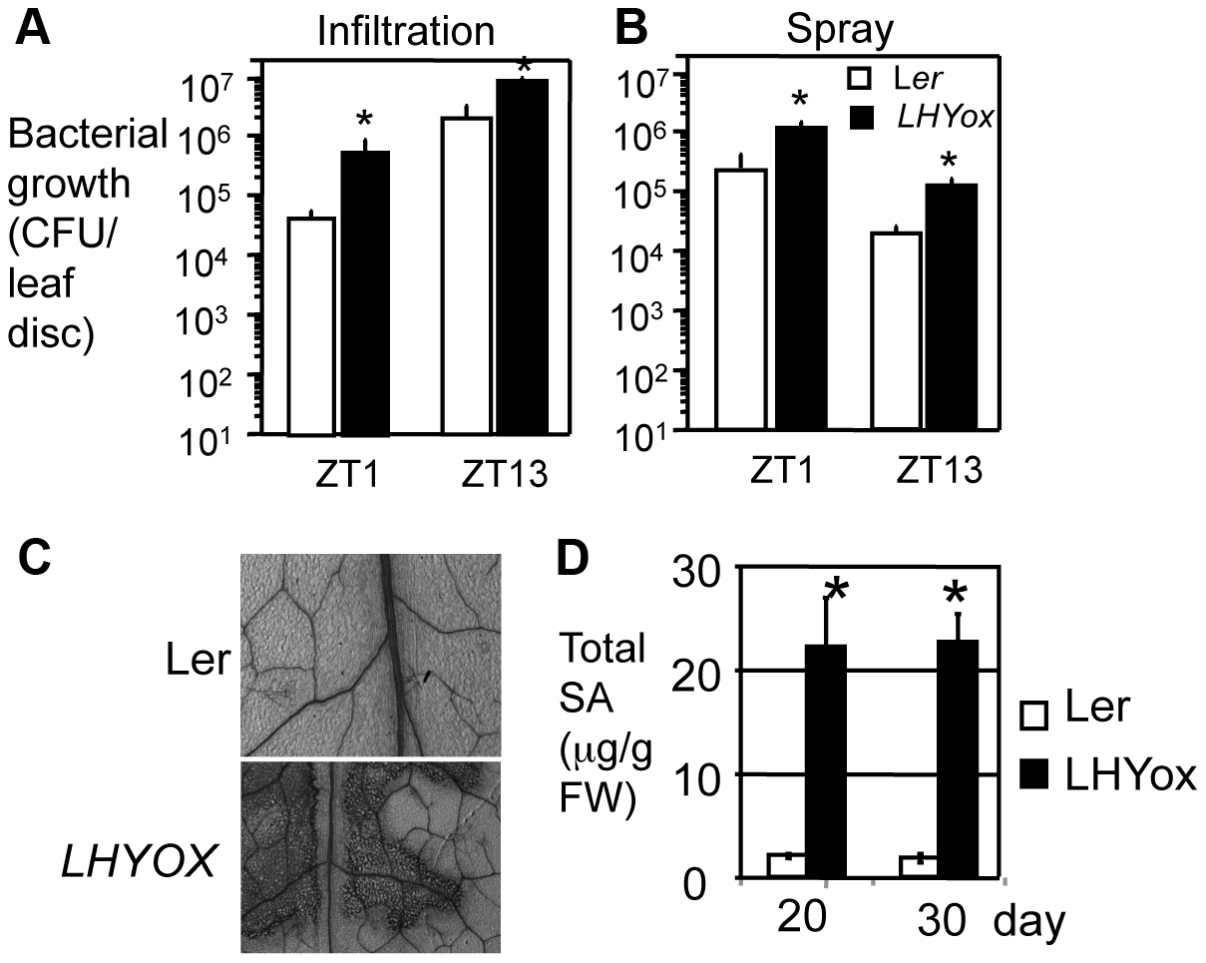
Overexpression of the *LHY* gene confers enhanced disease susceptibility to *P.* *syringae*. (**A**) Infiltration with DC3000. (**B**) Spray with DC3000. 30-day-old plants were infected with *P. syringae pv. tomato* strain DC3000 (DC3000) by infiltration (1×10^5^ CFU/ml) or spray (1×10^8^ CFU/ml) at ZT1 or ZT13 in LD. Bacterial growth was assessed at 3 dpi. (**C**) Cell death staining. The fourth to fifth leaves of Ler and *LHYox* were stained with trypan blue to visualize cell death [54]. (**D**) SA quantification. Total SA was extracted from 20- and 30-day old plants. Data represent the average of SA levels (n = 3) ± standard deviation. Statistical analysis was performed with Student's t-test (StatView 5.0.1). Asterisks indicate significant difference between L*er* and *LHYox* at the same time point (P<0.05). These experiments were repeated three times with similar results.

*LHYox* plants are in the Landsberg *erecta* (L*er*) background, with which we used *P. syringae pv. tomato* DC3000 (DC3000) to test disease resistance because this strain induces stronger disease symptoms in our hands than does *PmaDG3*. Similar to *CCA1ox* plants, *LHYox* plants had more bacterial growth than L*er* when infiltrated with DC3000 at ZT1 or ZT13 in LD (Figure 4A). In addition, spray-infection at ZT1 or ZT13 in LD also gave similar results (Figure 4B). Together, disruption of the circadian clock by misexpressing *CCA1* and/or *LHY* compromises disease resistance to *P. syringae*, supporting a direct role of the circadian clock in defense regulation.

### CCA1 and LHY gate stomatal response to dark and to *P. syringae* infection

**Figure 5:**
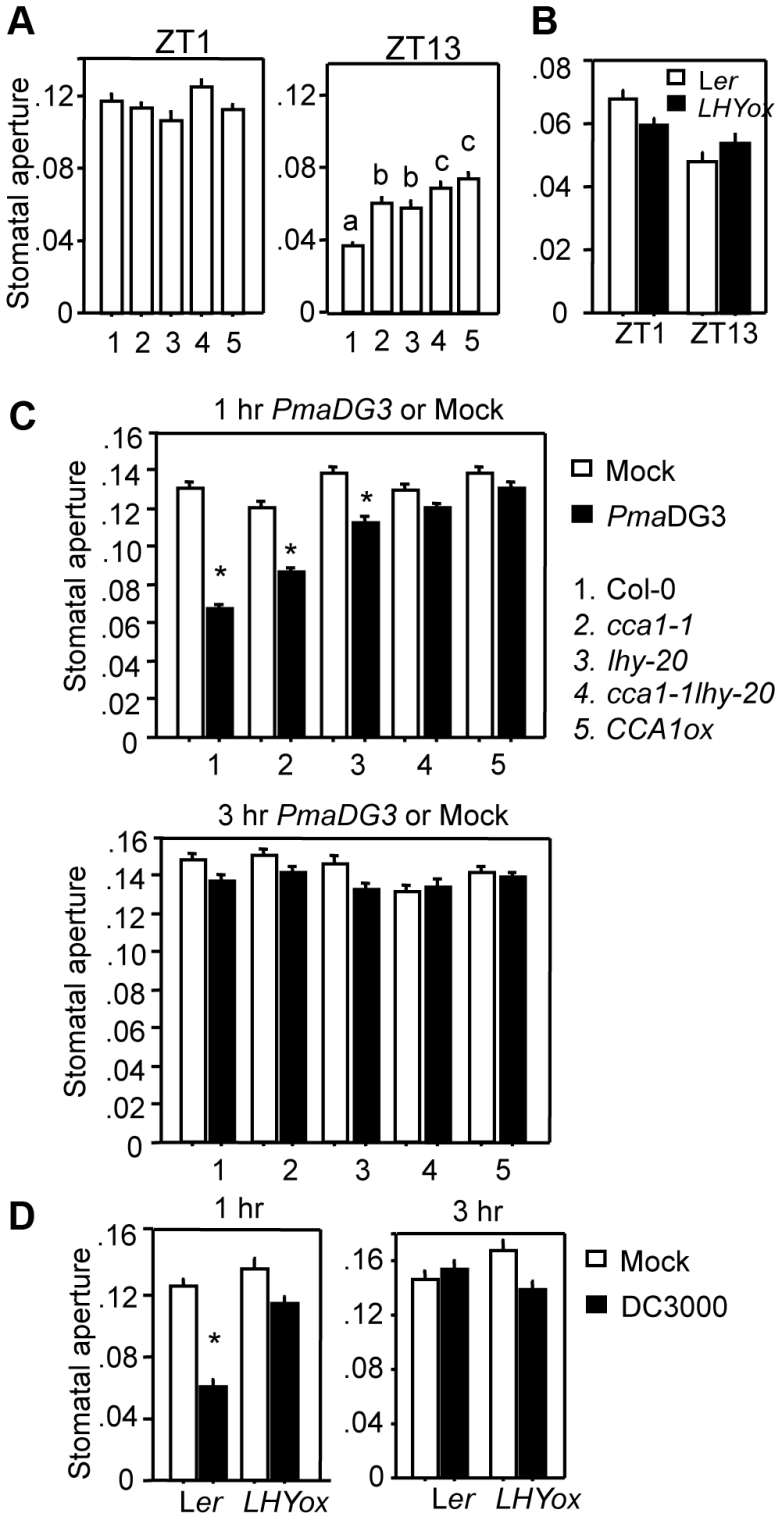
Disruption of *CCA1* and *LHY* leads to altered stomatal activity. (**A**) Stomatal aperture at ZT1 (left) or ZT13 (right) for Col-0, *cca1-1*, *lhy-20*, *cca1-1lhy-20*, and *CCA1ox*. (**B**) Stomatal aperture at ZT1 or ZT13 for L*er* and *LHYox*. (**C**) Stomatal aperture at 1 hr (top) or 3 hr (bottom) after exposure to *PmaDG3* or mock for Col-0, *cca1-1*, *lhy-20*, *cca1-1lhy-20*, and *CCA1ox*. (**D**) Stomatal aperture at 1 hr or 3 hr after exposure to *DC3000* or mock for L*er* and *LHYox*. For (**A**) and (**B**), three leaves from uninfected 25-day-old plants grown in 12 hr light/12 hr dark at 22°C were taken at the indicated times for the measurement of stomatal aperture. For (**C**) and (**D**), *P. syringae* treatment was conducted at ZT4 to ensure that most stomata were open upon treatment. Leaves were immersed in bacterial suspension (10^8^ cfu/ml) or water as mock treatment. At least three leaves of a genotype were collected at the indicated times for stomatal aperture measurement. Data represents the average of three experiments ± SEM. Each of these experiments contains at least 70 randomly chosen stomata. Different letters in (**A**) indicate significant difference among the samples. Asterisks in (**C**) and (**D**) indicate significant difference between mock-treated and infected plants of the same genotype (P<0.001; Student's t-test). These experiments were repeated three times with similar results.

Our data show that *cca1-1lhy-20* was more susceptible with spray-infection and *CCA1ox* and *LHYox* plants displayed enhanced susceptibility with both spray and infiltration infections. These suggest that both stomata-dependent and -independent defense can be affected by misexpression of either of these two core oscillator genes. Consistent with this notion, a previous study showed that *CCA1ox* plants had increased CO_2_ assimilation and stomatal conductance [13]. To further test whether the defense role of CCA1 and LHY is linked to the control of stomatal pore size, we measured plant stomatal aperture at ZT1 and ZT13 in LD. Consistent with Col-0 being more resistant with spray-infection at ZT13 than at ZT1, we found that stomatal aperture of Col-0 was much smaller at ZT13 than at ZT1 (Figure 5A). Compared with Col-0, the *cca1-1* and *lhy-20* mutants and *CCA1ox* plants showed similar stomatal aperture at ZT1 but had greater stomatal aperture at ZT13 (Figure 5A). These data suggest that disrupting clock activity mediated by CCA1 and LHY could make plants less responsive to dark-induced stomatal closure at night, thereby enhancing access of *P. syringae* to the leaf interior.

To further determine how these mutants respond to *P. syringae* infection, we measured stomatal aperture in the presence of *PmaDG3*. *PmaDG3* treatment was performed at ZT4 after plants had been exposed to light for four hours to ensure the opening of the stomata (Figure S3). At 1 hr post infection (1 hpi), we observed a 48.1% suppression of stomatal aperture in Col-0, compared with mock treatment (Figure 5C top and Table S1). However, this suppression was much reduced in *cca1-1* and *lhy-20* and largely blocked in *cca1-1lhy-20* and *CCA1ox*. *P. syringae*-induced stomatal closure was transient since both mock and *PmaDG3-*treated leaves showed similar stomatal aperture at 3 hpi (Figure 5C bottom). Although exhibiting similar stomatal aperture at ZT1 and ZT13 (Figure 5B), the *LHYox* plants also showed reduced suppression of DC3000-induced stomatal closure at 1 hpi (16.9%), compared with L*er* control (51.6%) (Figure 5D and Table S1). Hence, these results indicate that disrupting the circadian clock by *CCA1* and *LHY* misexpression impairs plants' capacity of inducing stomatal closure in response to *P. syringae*.

### CCA1 and LHY contribute synergistically to *Hpa* resistance

**Figure 6:**
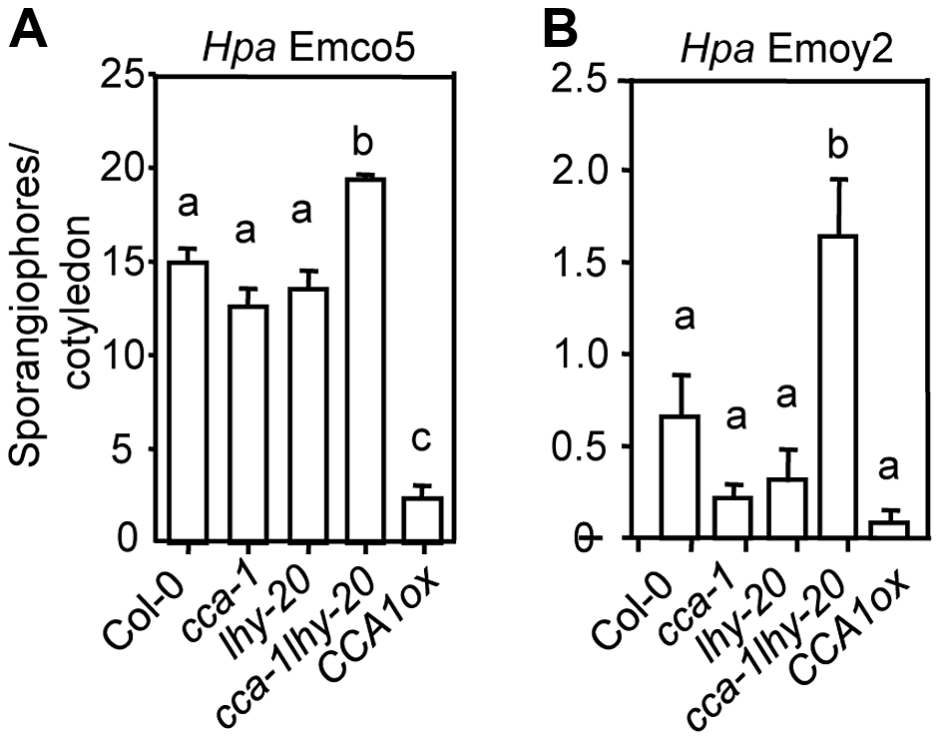
CCA1 and LHY contribute synergistically to resistance to *Hyaloperonospora arabidopsidis* (*Hpa*). (**A**) Infection with *Hpa* Emco5. (**B**) Infection with *Hpa* Emoy2. Seven-day-old seedlings were spray-infected at ZT7 in LD with the virulent strain *Hpa* Emco5 or the avirulent strain *Hpa Emoy2* (5×10^4^ spores/ml in water). Sporangiophore production in cotyledons of each genotype was counted at 7 dpi. Data represent the average number of sporangiophores from 20 seedlings for *CCA1ox* and 50 seedlings for other genotypes ± SEM. Letters indicate significant difference among the samples (P<0.01; Mann-Whitney test). These experiments were repeated three times with similar results.

CCA1 but not LHY was previously shown to regulate resistance to the oomycete pathogen *Hpa* [28]. To test whether a contribution of LHY to *Hpa* resistance could be discerned in the double mutant *cca1-1lhy-20*, we sprayed seven-day-old seedlings at ZT7 in LD with the virulent strain *Hpa* Emco5 or the avirulent strain *Hpa* Emoy2 (recognized by the R protein RPP4 in Col-0). We observed significantly more susceptibility to both *Hpa* strains in the *cca1-1lhy-20* double mutant, compared to Col-0 and the single mutants (Figure 6A and 6B) while the *CCA1ox* plants were substantially more resistant to *Hpa* Emco5 (Figure 6A). Our data are broadly in agreement with those previously reported [28]. The reason that we did not observe a significant difference between Col-0 and *cca1-1* could be due to the difference in the infection time and/or *Hpa* strains used - Wang et al inoculated plants with the avirulent strain *Hpa* Emwa1 at dawn [28] while we used *Hpa* Emco5 (virulent) and Emoy2 (avirulent) in the afternoon in our experiments. Nevertheless, these data, together with the *P. syringae* data described earlier, demonstrate that CCA1 and LHY contribute synergistically to basal resistance and *R*-gene mediated defense against both bacterial and oomycete pathogens. What surprises us is the difference in response to *P. syringae* (decreased resistance) and *Hpa* (enhanced resistance) strains observed in *CCA1ox* plants. We speculate that there are distinct mechanisms that these plants use to defend against the two pathogens.

### Defense-related genes might be preferentially regulated by CCA1 and LHY

**Figure 7:**
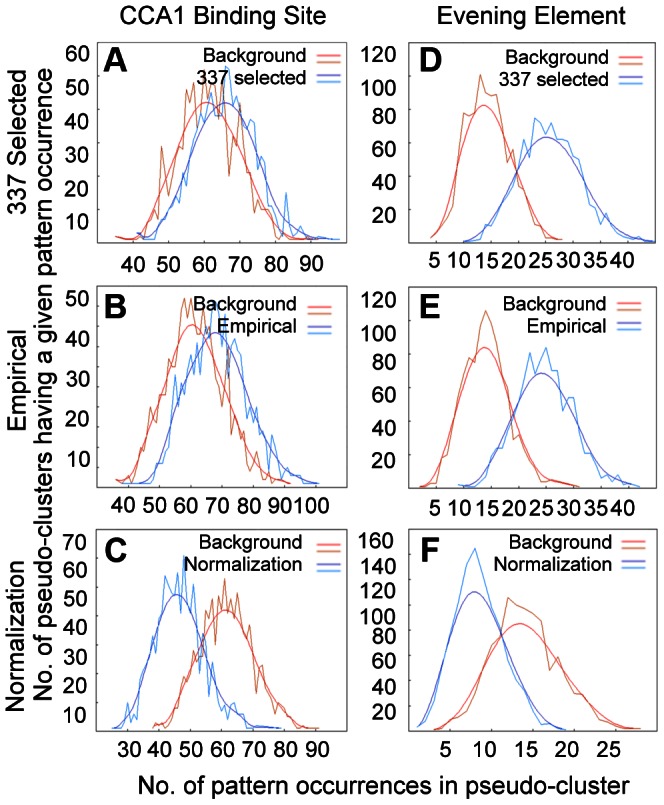
Motif enrichment analysis of 571 gene promoters. A total of 571 promoters from genes in three categories, selected (337 defense-related gene based on microarray experiments), empirical (127 empirical marker genes for various pathogen responses), and normalization (107 non-defense related genes) [43], was analyzed for the enrichment of CBS or EE motifs, using the online tool POBO (http://ekhidna.biocenter.helsinki.fi/poxo/pobo/) [44]. (**A**) and (**D**) are for selected genes, (**B**) and (**E**) are for empirical genes, and (**C**) and (**F**) are for normalization genes. Panels (**A**), (**B**), and (**C**) are for the CBS motif and panels (**D**), (**E**), and (**F**) are for the EE motif. The red lines represent the background while the blue lines represent one of three sets of genes used in each analysis.

Identification of defense-related genes controlled by CCA1 and LHY is critical to gain better understanding of the mechanism of action of CCA1 and LHY in defense regulation. To this end, we analyzed promoters of 571 genes for CCA1-binding site (CBS) and evening element (EE), two *cis* elements known for CCA1 and LHY binding [40], [41], [42]. These 571 genes had been previously selected to construct mini-microarrays, consisting of three groups, selected (337 defense-related genes based on microarray experiments), empirical (127 empirical marker genes for various pathogen responses), and normalization (107 non-defense related genes whose expression levels were relatively stable among experiments with pathogen infection) [43]. The online tool POBO [44] was used to analyze up to 3000 bp from the promoter regions of these genes, which do not include the coding sequences of neighboring genes, for an enrichment of CBS or EE motifs. The background for this analysis was generated using pseudo-clusters of 100 promoters of up to 3000 bp in length of randomly sampled Arabidopsis genes (1000 bootstrap replications were used in the sampling). Compared with the background, the CBS motif was found as often as expected by chance in the selected and empirical gene promoters (Figure 7A and 7B) but the motifs were found less frequently in the normalization gene promoters (Figure 7C and Table S2). When compared to the normalization genes, there was a greater than 40% increase of the cluster mean for the CBS motif in both selected and empirical genes. These observations suggest that although defense-related genes (selected and empirical genes) are not particularly enriched with the CBS motif, the non-defense related genes (the normalization genes) are slightly depleted of the motif. The enrichment of the EE motif was more pronounced in both selected and empirical genes, with about 200% increase of the cluster means when compared to the normalization genes (Figure 7D–7F and Table S2). Thus, these results suggest that defense-related genes are preferentially regulated by CCA1 and LHY. However, since the sample size in each group is small, caution should be taken when extrapolating this interpretation to the whole genome level.

### The defense gene *GRP7* acts downstream of CCA1 and LHY

**Figure 8:**
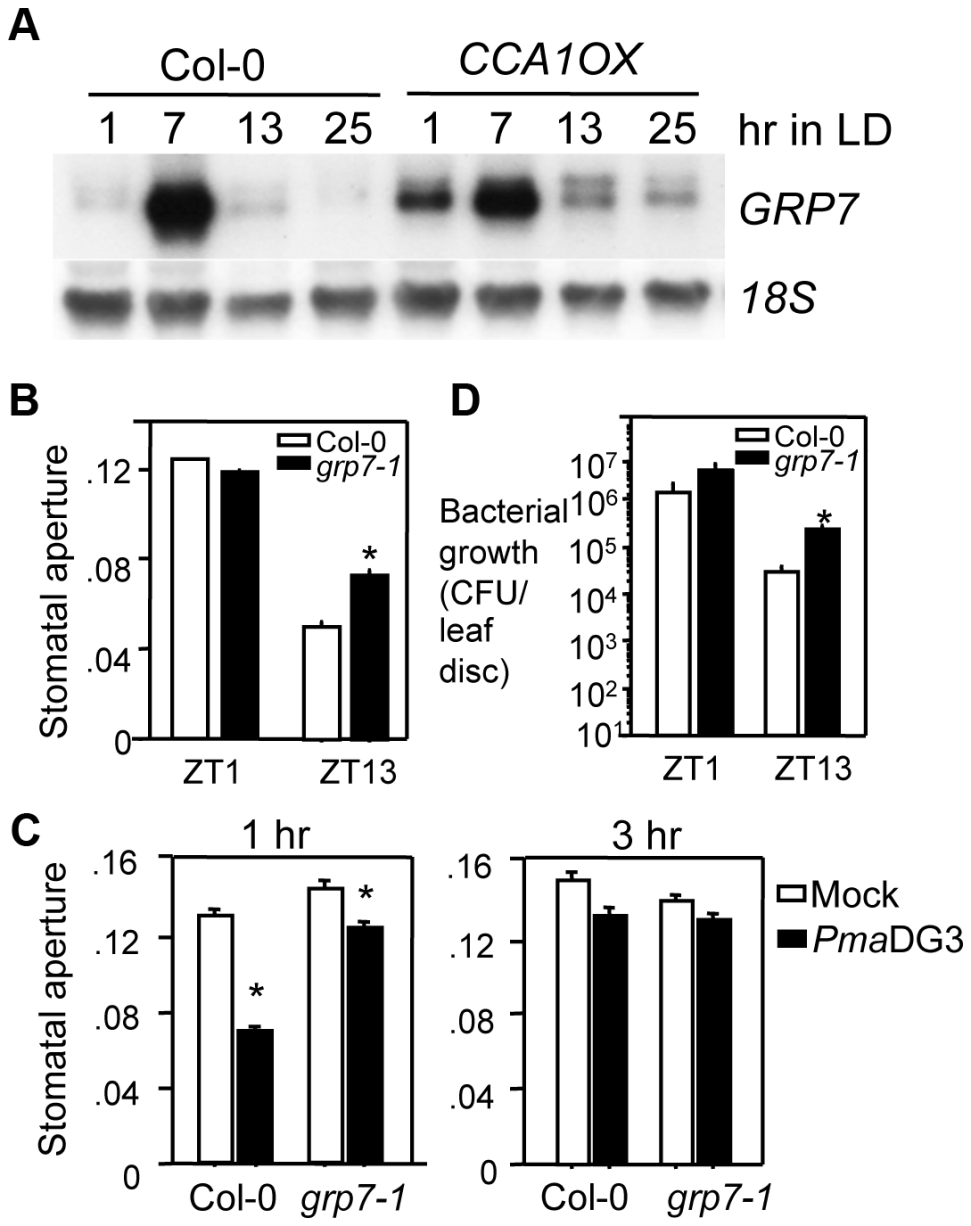
CCA1-regulated *GRP7* affects disease resistance to *P.* *syringae* and stomatal activity. (**A**) Expression of *GRP7* is disrupted in *CCA1ox* in LD. Twenty five-day-old Col-0 and *CCA1ox* plants grown in a chamber with a 12 hr light/12 hr dark cycle and 22°C were harvested at ZT1 at 6 hr interval for 24 hrs followed by RNA extraction and northern blotting. 18S rRNA was used as a loading control. These experiments were repeated twice with similar results. (**B**) Stomatal aperture at ZT1 or ZT13. (**C**) Stomatal aperture at 1 hr (left) or 3 hr (right) after exposure to *PmaDG3* or mock solution. (**D**) Bacterial growth assay with ZT1 or ZT13 infection in LD. Asterisks indicate significant difference among the samples at the indicated times in panels (**B**) and (**D**) or within the same genotypes in panel (**C**) (P<0.05; Student's t-test). These experiments were repeated twice with similar results.

The frequency of CBS or EE motif per promoter region was quantified from the above three sets of genes (Figure S4). Among the genes analyzed, we found that *GRP7* (At2g21660; also known as *COLD AND CIRCADIAN REGULATED 2* [*CCR2*]) [45], [46] had the most overrepresentation of the EE motif, with four EE within a 300 bp promoter region. One CBS motif was also found at 1294 bp of the *GRP7* promoter. GRP7 is a key constituent of a slave oscillator regulated by the circadian clock [45], [47] and also has been demonstrated to have roles in regulating floral transition and plant defense [48], [49]. Expression of *GRP7* was previously shown to be circadian regulated with a shortened circadian period in a *cca1lhy* double mutant and a disrupted pattern in *CCA1ox* plants [12], [20], [50]. However, *GRP7* had never been explicitly established as a target gene of CCA1 and LHY. Our northern analysis confirmed circadian expression of *GRP7* and showed that such expression was slightly affected by the *cca1-1* mutation and became arrhythmic in *CCA1ox* in LL (Figure S5). We also observed disrupted expression of *GRP7* in *CCA1ox* plants in LD (Figure 8A). Thus, these data further confirm that *GRP7* is regulated by CCA1.

GRP7 was previously demonstrated to regulate stomatal activity [51]. We found that similar to *cca1-1lhy-20* and *CCA1ox* plants, stomatal aperture of *grp7-1* was greater than that of Col-0 at ZT13 (Figure 8B). In response to *PmaDG3* infection, *grp7-1* displayed 14.2% suppression of stomatal aperture whereas Col-0 showed 48.1% suppression at 1 hpi (Figure 8C, S3, and Table S1), suggesting that *grp7-1* has reduced responsiveness to *PmaDG3* in stomatal closure. We further found that *grp7-1* was significantly more susceptible to *PmaDG3* than Col-0 when spray-infected at ZT13 in LD (Figure 8D). Together our bioinformatic analysis and experimental evidence indicate that *GRP7* is a target of CCA1 and/or LHY that regulates stomatal activity and modulates plant defense.

### CCA1 and LHY regulate disease resistance independently of SA

**Figure 9:**
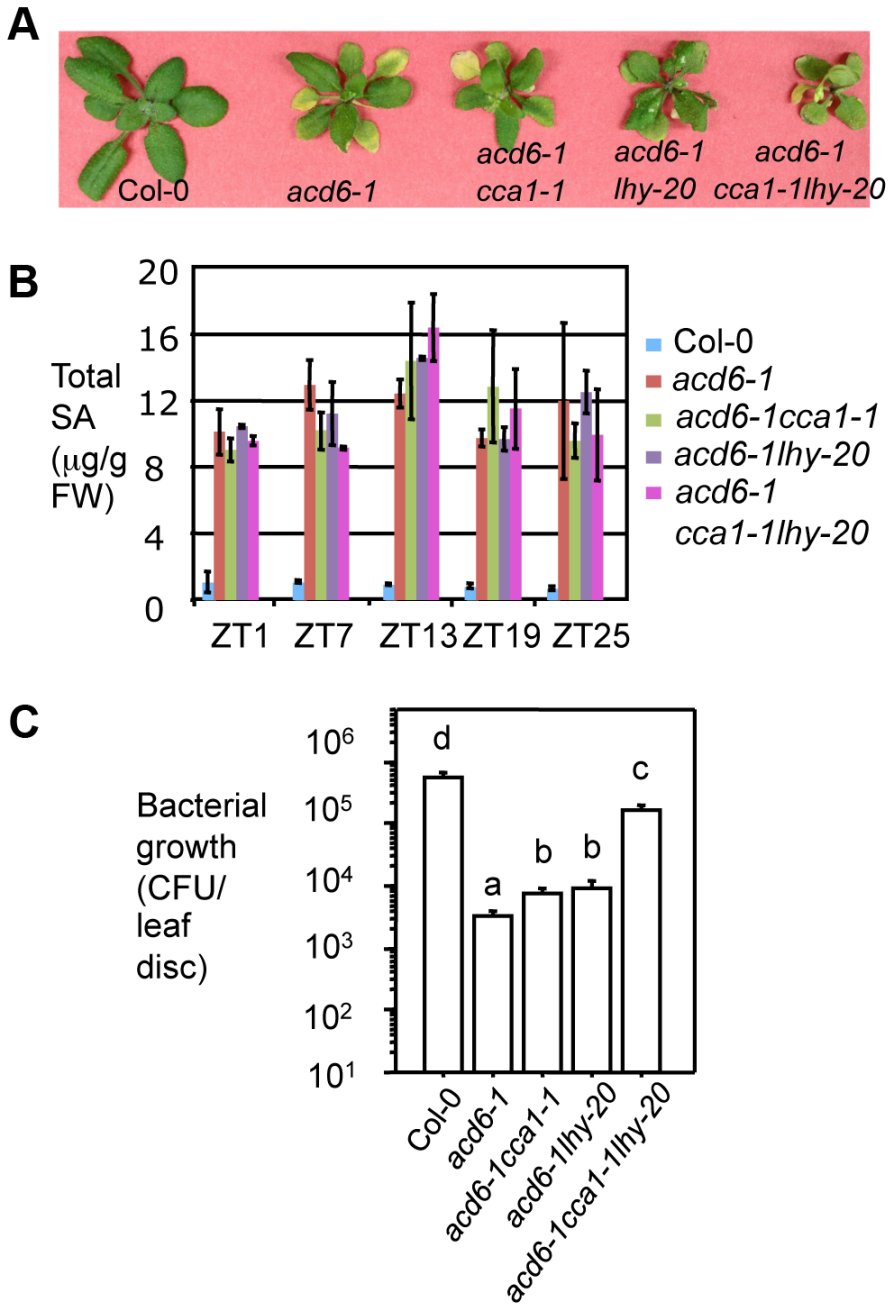
CCA1 and LHY conferred disease resistance is SA-independent. (**A**) Picture of 25-day-old plants. (**B**) SA quantitation. Twenty-five-day old plants grown in 12 hr light/12 hr dark cycle (LD) at 22°C were harvested at ZT1, 7, 13, 19 and 25. Total SA were extracted and measured as described [90]. (**C**) Infection with *PmaDG3*. Twenty five-day-old plants were infected by spraying with the virulent strain *PmaDG3* (1×10^8^ CFU/ml) at ZT13. Bacterial growth was assessed at 3 dpi. Data represent the mean bacterial numbers ± SEM (n = 6). Letters indicate significant difference among the samples (P<0.05; Student's t-test). These experiments were repeated three times with similar results.

SA is a key signaling molecule involved in both basal resistance and *R* gene-mediated defense. The *accelerated cell death 6-1* (*acd6-1*) mutant shows constitutive defense, high levels of SA, and extremely small size that is sensitized to the change of SA defense [52], [53]. Thus, *acd6-1* has been used as a convenient readout to gauge the effect of some known defense genes in regulating SA-mediated defense [54], [55], [56]. To determine whether CCA1 and LHY act through SA, we crossed *cca1-1lhy-20* to *acd6-1* and obtained homozygous double (*acd6-1cca1-1* and *acd6-1lhy-20*) and triple (*acd6-1cca1-1lhy-20*) mutants. We found that both double and triple mutants resembled *acd6-1*, displaying dwarfism and accumulating similar SA levels (Figure 9A and B). However, when spray-infected with *PmaDG3* at ZT13, the double mutants were slightly more susceptible while the triple mutant was much more susceptible than *acd6-1* (Figure 9C). These results corroborate a synergistic interaction between CCA1 and LHY in clock and defense regulation. They also suggest that the defense role of CCA1 and LHY is largely SA-independent. Consistent with this notion, we found that in the absence of *acd6-1*, the SA levels are comparable among Col-0, *cca1-1*, *lhy-*20, *cca1-1lhy-20*, and *CCA1ox* in LD (Figure S6A). In addition, although more susceptible to *P. syringae* infection, *LHYox* plants were dwarf, showed spontaneous cell death, and accumulated high levels of SA (Figure 4C, 4D, and S6B). Together, these results indicate that CCA1 and LHY act independently of SA to regulate resistance to *P. syringae*.

### Defense activation reciprocally regulates clock activity

**Figure 10:**
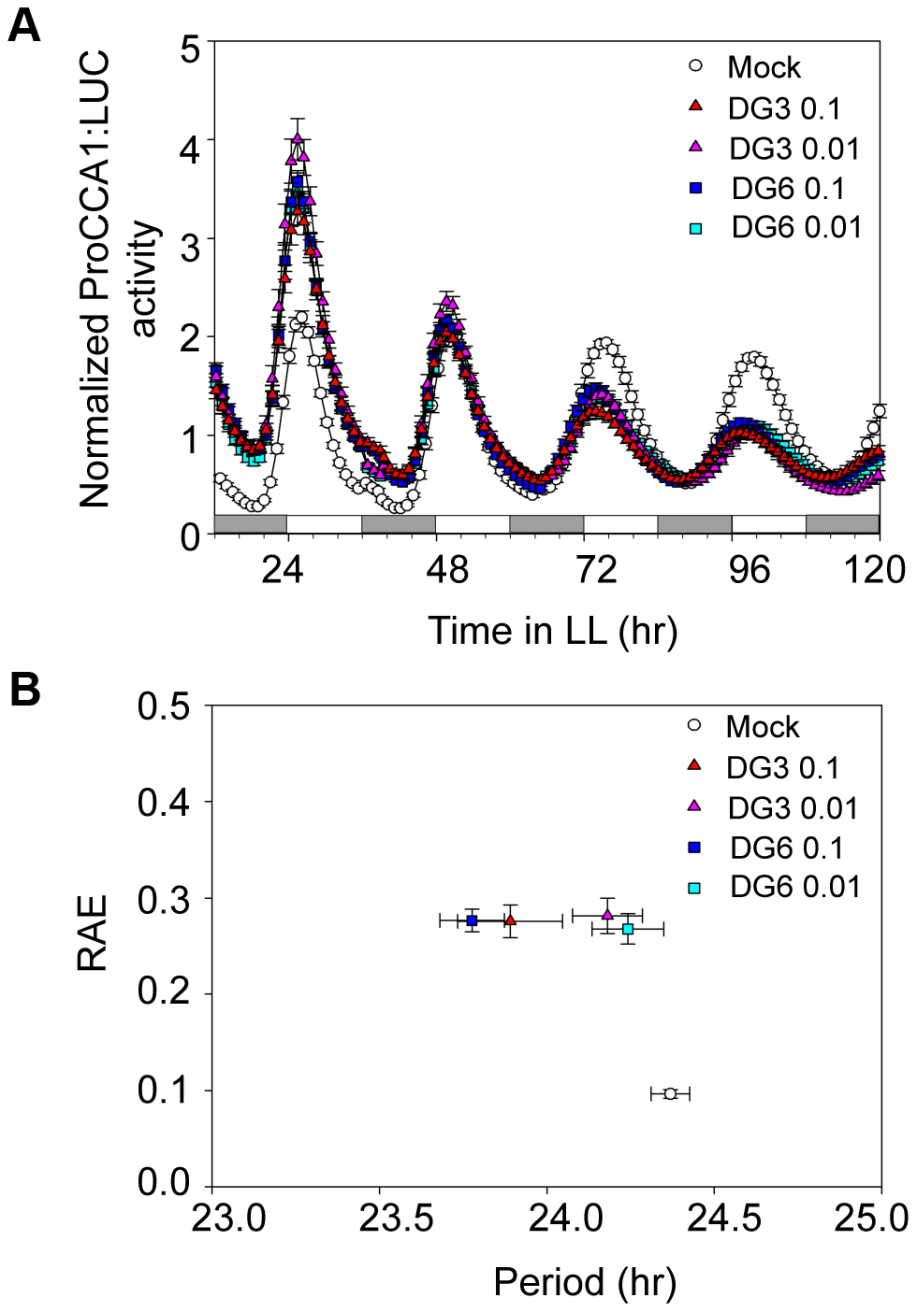
Defense activation by *P.* *syringae* infection shortens the period of the *ProCCA1:LUC* reporter activity. (**A**) Mean circadian traces for *ProCCA1:LUC* activity. (**B**) Mean circadian period of the *ProCCA1:LUC* reporter. Col-0 seedlings expressing the *ProCCA1:LUC* reporter were grown from germination in 12 hr light/12 hr dark cycles at 22°C. At ZT7, eight-day-old seedlings were incubated with *PmaDG3* or *PmaDG6* (1×10^8^ or 1×10^7^ CFU/ml, labeled as 0.1 or 0.01, respectively) for 3 mins, blot dried, and transferred to 96-well plates containing 200 µl of MS media and 30 µl of a 2.5 mM D-luciferin solution. Luciferase activity was recorded with a Packard TopCount luminometer in LL at 22°C. RAE: relative amplitude error. RAE values close to zero indicate strong rhythms while those close to 1 indicate the limit of statistically significant rhythmicity. SEM (n = 12–24) was used for (**A**) and (**B**). These experiments were repeated twice with similar results.

Our data and those from other groups clearly indicate that plant innate immunity is an output event regulated by the circadian clock. However, it is not known whether this regulatory relationship is reciprocal with defense activation feeding back to affect clock activity. To test this, we infected Col-0 expressing the *ProCCA1:LUC* reporter with both virulent and avirulent *P. syringae* strains. Bioluminescence analysis indicated that the period of *ProCCA1:LUC* was significantly shortened in the presence of the virulent strain *PmaDG3* or the avirulent strain *PmaDG6* at a high dose (OD = 0.1) (Figure 10 and Table S3). Similarly, infection of Col-0 seedlings expressing *ProGRP7:LUC* also resulted in period shortening of *ProGRP7*-controlled luciferase activity (Figure S7 and Table S3). These results suggest that clock activity is modulated by both basal and RPS2-mediated defenses.

**Figure 11:**
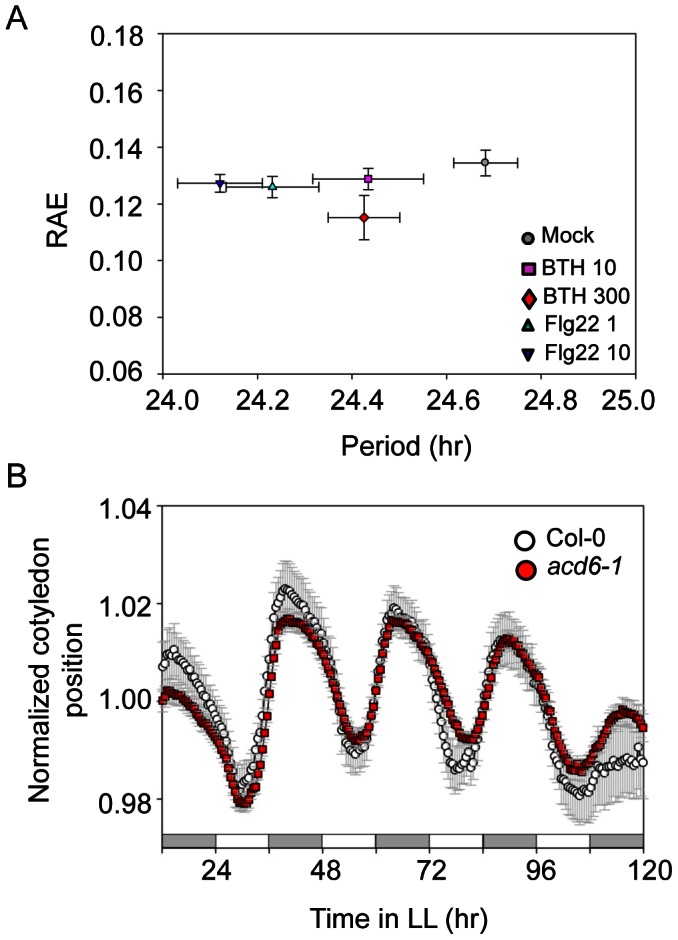
The clock period is shortened by treatment with flg22 but not with BTH. (**A**) Mean circadian period of the *ProCCA1:LUC* reporter. Eight-day-old Col-0 seedlings expressing the *ProCCA1:LUC* reporter were grown from germination in 12 hr light/12 hr dark cycles at 22°C. At ZT7, eight-day-old seedlings were treated with flg22 (1 µM or 10 µM) or BTH (10 µM or 300 µM) and transferred to 96-well plates containing 200 µl of MS media and 30 µl of a 2.5 mM D-luciferin solution. Luciferase activity was recorded with a Packard TopCount luminometer in LL at 22°C. (**B**) Cotyledon movement assay with *acd6-1*. Eight-day-old *acd6-1* seedlings grown in a 12 hr light/121 hr dark cycle at 22°C were transferred to 24-well cloning plates and recorded in LL at 22°C for cotyledon movement. SEM (n = 12–24) was used for (**A**) and (**B**). These experiments were repeated twice with similar results.

To further investigate which defense signaling pathway(s) are involved in the feedback-regulation of clock activity, we treated Col-0/*ProCCA1:LUC* seedlings with flg22 or benzo (1,2,3) thiadiazole-7-carbothioic acid (BTH). Flg22 is a 22-aa synthetic peptide from the conserved region of flagellin proteins of *P. syringae* and elicits plant basal defense in a wide variety of plant species [4], [57]. BTH is an agonist of SA that efficiently activates SA signaling [58]. We found that flg22 at both doses (1 µM and 10 µM) significantly shortened the period of *CCA1* expression. However, BTH treatment (10 µM and 300 µM) did not change *CCA1* promoter activity (Figure 11A and Table S3). To further test if SA could affect clock activity, we used a cotyledon movement assay [59] to gauge clock activity in the *acd6-1* mutant, which constitutively accumulates high levels of SA [52], [53]. We found that *acd6-1* showed similar period, phase, and amplitude of the rhythm for cotyledon movement to Col-0 (Figure 11B and S8). Taken together, these data indicate that activation of flg22-triggered basal defense but not SA signaling can feedback to regulate clock activity.

## Discussion

Increasing evidence has implicated a role of the circadian clock in regulating plant innate immunity. Of the components in the central oscillator of the circadian clock, CCA1 is the first shown to affect plant defense against *P. syringae* and *Hpa* [27], [28]. However, its close homolog, LHY, has not been shown such a role, despite the fact that loss-of-function mutants in both genes displayed similarly shortened period. Thus, it was unclear whether plant innate immunity is regulated by the circadian clock mediated by CCA1 or by non-clock related function of CCA1. Here we show that disrupting clock function by misexpression of *CCA1* and/or *LHY* leads to compromised immunity, thus further establishing a direct role of the circadian clock in defense regulation. Our data suggest that one of the mechanisms by which CCA1 and LHY regulate plant innate immunity is through affecting stomatal defense with the downstream target gene *GRP7*. We further demonstrate that defense activation by *P. syringae* infection and flg22 treatment shortens circadian period. Thus this study reveals for the first time crosstalk between the circadian clock and plant innate immunity.

### The circadian clock mediated by CCA1 and LHY regulates plant defense under LL and LD conditions

Typical studies of the circadian clock have been performed under constant light (LL) conditions to emphasize the endogenous nature of the clock. In LL, perturbations of the circadian clock typically result in altered period length; for instance, loss of CCA1 or LHY function shortens circadian period. However, plants typically grow in LD cycles in which the environmental cycle entrains even a mutant clock to a 24-hour period. Under such LD conditions, perturbations in the circadian clock can manifest as alterations in phase for reporter gene expression (Figure 1 and S1B and [20], [21]) as well as changes in a variety of other traits, including flowering time, metabolism, stomatal activity, gene expression patterns, and defense responses [12], [13], [20], [29], [50], [60], [61]. Thus, the effects of disrupted circadian clock could become apparent under LL and LD conditions.

Several studies indicate that the circadian clock mediated by CCA1 and LHY regulates plant defense in both LL and LD ([27], [28] and this study). For instance, Bhardwaj et. al. showed that *CCA1ox* plants were more susceptible to *P. syringe* infection than wt in LL [27]. Here we extend this observation by showing that *CCA1ox* plants had enhanced susceptibility to *P. syringae* in both LL and LD (Figure 2, 3, and S2). In LD, enhanced susceptibility was also observed in *cca1-1lhy-20* and *LHYox* plants to *P. syringae* strains (Figure 3 and 4) and in *cca1-1lhy-20* to *Hpa* strains (Figure 6). Consistent with our data, a single *cca1* mutant showed compromised resistance to a different *Hpa* strain and affected expression of some defense-related genes in LD [28]. Together these studies firmly establish that plant innate immunity is an output regulated by the circadian clock under LL and LD conditions.

While we mainly focus our analyses in this report on defense phenotypes regulated by CCA1 and LHY in LD, we also agree that we should use caution when interpreting our results since the effect of the circadian clock can manifest differently under different light conditions, including both differing daylengths and light intensities. For instance, it is possible that the degree of susceptibility to pathogen infection and the severity of stomatal change in response to dark and *P. syringae* infection could be different in LD from those in LL in *cca1-1lhy-20* (compared with wt). Alternatively, the amplitude, period, and/or phase of defense gene expression could be different in *cca1-1lhy-20* (compared with wt) in LD from those in LL. Even different LD conditions could have different effects on clock activity. For example, Michael et al. [62] showed that the set of cycling transcripts increased with the number of different cycling conditions examined. We found that in 12 hr L/12 hr D, expression of *GRP7* retained rhythmicity in *CCA1ox*, compared with that in wt, although the waveform was altered with baseline expression increased (Figure 8A). However, Green et al observed more pronounced alterations in phase of *GRP7* expression in *CCA1ox* (compared with that in wt) in seedlings growing in long or short daylengths (16 hr L/8 hr D or 8 hr L/16 hr D), with maximal transcript accumulation in the dark [12]. Such differences in the patterns of *GRP7* transcript abundance could also be due to other reasons besides light conditions. Nonetheless, these observations together with those of Michael et al. [62] emphasize that to better understand the role of the circadian clock in defense control, analyses of defense phenotypes with plants misexpressing *CCA1*, *LHY*, and/or other clock genes should be carried out in LL, DD, and different LD conditions for a comprehensive comparison.

### Plants employ different mechanisms to defend against pathogens at different times of day

Although encountering pathogens at different times in a day, Arabidopsis plants were suggested to be more resistant in the morning than at night. To support this conclusion, wt plants demonstrated higher resistance and/or defense responses when infiltrated during the day than at night [27], [63]. We also observed similar results in plants infiltrated with *P. syringae* in LL or LD (Figure 2, 4A, and S2), thus supporting this conclusion. However, with spray-infection in LD, we observed the opposite phenotype; wt plants were more resistant at night than in the morning (Figure 3, 4B and 8D). During spray-infection, *P. syringae* initially lands on the leaf surface. Further invasion depends on the success of the bacteria in gaining entry into the host tissue via natural openings, such as stomata [1], [2]. Consistent with enhanced disease resistance to sprayed *P. syringae*, plants in the evening have much smaller stomatal pore sizes than in the morning (Figure 5A, 5B, and 8B).

**Figure 12:**
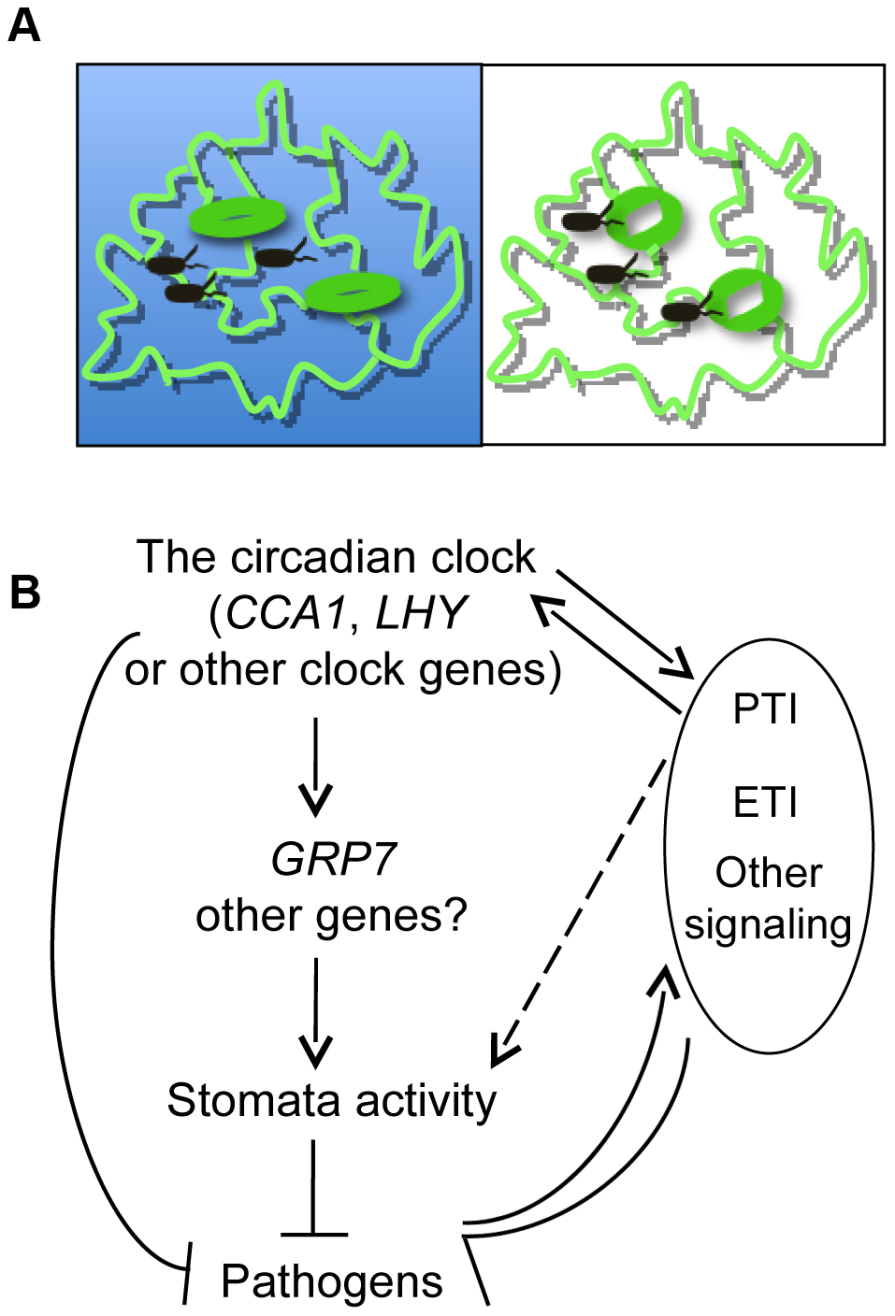
A simplified model for crosstalk between the circadian clock and plant innate immunity. (**A**) Timing of stomata-dependent and -independent defense in a day. At night, plants might rely more on closed stomata to provide physical constrains to limit pathogen invasion but have relatively lower levels of stomata-independent defense. Once pathogens bypass such constrains (i.e. via infiltration infection in the laboratory), they encounter a plant host that is more susceptible than during the day. During the day, most stomata are wide open. In the presence of pathogens, plants can only transiently reduce stomatal aperture for a few hours (this study and [1]). Thus, during the day plants might depend more on stomata-independent defense to restrict pathogen invasion. Stomata-dependent defense could be stronger at night while stomata-independent defense could be stronger during the day. (**B**) The circadian clock regulates both stomata-dependent and -independent defense pathways to restrict pathogen growth in Arabidopsis. In a stomata-dependent pathway, CCA1 and LHY act, at least in part, through GRP7 as a direct downstream target to regulate stomatal aperture and thereby defense. Other downstream targets of CCA1 and LHY and other components of the central oscillator of the circadian clock might also be involved in regulating stomata-dependent and –independent defense. On the other hand, pathogen infection can activate PTI, ETI and other defense signaling in the host. PTI induced by flg22 feeds back to regulate clock activity. In addition, flg22-triggered signaling is under circadian clock control [27]. Thus, we conclude that the clock-defense crosstalk involves flg22-mediated signaling. Flg22 can affect stomatal aperture [91]. However, whether this function of flg22 is through its regulation of the circadian clock or through a direct regulation of stomata is unclear. Other questions, such as whether additional PAMPs, effectors, and other defense signaling molecules are involved in clock-defense crosstalk, remain to be answered.

These two seemly contradicting results actually coalesce to suggest different mechanisms that plants use to defend against pathogens at different times of day, depending on the mode of pathogen invasion. As summarized in Figure 12A, at night plants might rely more on closed stomata to physically restrict pathogen invasion but stomata-independent defense is relatively low. If a pathogen can breach stomatal restriction (i.e. being pressured into host tissue via infiltration in the laboratory) at night, it can be more virulent to the host. However, with stomata widely open during the day, plants apparently compensate for enhanced pathogen access to the leaf interior with enhanced stomata-independent defense that is stronger during the day than at night. This cycling in host resistance means that plants can be more resistant to epiphytic pathogens at night than during the day. But in the presence of apoplastic pathogens, plants can activate stronger defense during the day than at night. Taken together, we conclude that plants rely on distinct mechanisms, involving stomata-dependent and stomata-independent defenses, to respond to pathogen attacks at different times of day.

### The circadian clock acts through stomata-dependent and -independent pathways to regulate defense

Our data suggest that both stomata-dependent and -independent defense can be affected by CCA1, LHY, and its downstream target GRP7. Consistent with such a role of CCA1 and GRP7, these proteins are expressed in guard cells [51]. It is conceivable that CCA1 and/or LHY proteins directly affect the abundance of GRP7 via binding to its promoter at different times of day, which in turn regulates stomatal aperture and thereby stomatal defense (Figure 11B). Since both CCA1 and GRP7 proteins are also found in other cell types besides the guard cells [51], [64], it is possible that CCA1/LHY/GRP7 also contribute to stomata-independent defense.

*GRP7* is unlikely to be the only target of CCA1 and LHY to regulate pathogen defense. First, our bioinformatic analysis suggests that a number of defense genes besides *GRP7* might be preferentially regulated by CCA1 and LHY (Figure 7). And second, plants overexpressing *GRP7* are not more susceptible to *P. syringae* (J. Alfano and H. Kang, personal communications) while *CCA1ox* and *LHYox* plants are more susceptible to *P. syringae* (this study). Thus, CCA1 and LHY presumably act through multiple downstream target genes to regulate plant defense. Identification of these additional defense genes controlled by CCA1 and LHY should advance our understanding of the mechanisms by which the circadian clock regulates plant defense.

Rhythmic variation in stomatal aperture is known to be regulated by the circadian clock [13], [37], [65]. Besides *CCA1* and *LHY*, other genes encoding components of the central oscillator may also affect stomatal defense. For instance, a mutation in *EARLY FLOWERING 3* (*ELF3*) was recently shown to suppress stomatal closure and disease resistance [27], [66]. *ELF3* might act through the *FLOWERING LOCUS T* gene, which is highly expressed in stomata of the *elf3* mutant and has been shown to affect stomatal activity [66]. In addition, the *timing of cab expression1-1* (*toc1-1*) mutant also shows defects in stomatal aperture [59], [67]. It is tempting to speculate that *ELF3*-mediated defense is related to its role in stomatal control and *TOC1* could also contribute to plant defense. However, further experiments are necessary to validate these speculations. Nevertheless, these observations suggest that the circadian clock can influence stomatal activity and possibly also stomatal defense via different pathways (Figure 12B).

Stomata have been proposed as a critical battleground during plant-bacterium interactions [1], [2]. However, it is not known whether stomatal defense can also restrict the invasion of pathogens with different life styles from those of bacteria. The oomycete pathogen *Hpa* does not enter host organs through stomata; rather, germinating spores produce hyphae that penetrate between host epidermal cells and extend through the intracellular space in the mesophyll layer. However, near the end of the infection cycle, hyphal tips emerge through the stomata to the exterior of the leaf and then differentiate into spore-bearing structures [68], [69]. Thus, it is possible that host control of stomatal aperture could influence this stage of the life cycle. Although the role of stomata in defense against *Hpa* has not been well established, the fact that *cca1-1lhy-20* showed enhanced susceptibility to *Hpa* infection relative to the single mutants and wt suggests such a role of the circadian clock. Interestingly, while conferring enhanced disease susceptibility to *P. syringae*, *CCA1ox* heightened resistance to *Hpa* (Figure 6A and [28]), suggesting that *CCA1ox* plants employ different mechanisms to defend against these two pathogens. However, it is not clear whether the enhanced *Hpa* resistance conferred by *CCA1ox* is related to the circadian clock or to another function resulting from *CCA1* overexpression.

### Defense activation reciprocally regulates the circadian clock

While regulating multifaceted physiological activities of plants, the circadian clock can also be influenced by external signals, such as changes of light, temperature, hormones, and nutrients [32], [70], [71], [72], [73], [74]. Here we show that infection with both virulent and avirulent *P. syringae* strains shortens circadian period in Arabidopsis (Figure 10 and S7). We further found that such feedback regulation can be recapitulated with flg22 treatment (Figure 11A). Thus, defense activation can also serve as an input signal to regulate clock activity besides being an output of the circadian clock.

Since flg22-triggered callose deposition and expression of genes involved in flg22 sensing and signal transduction were previously shown to be under circadian clock control [27], we conclude that the clock-defense crosstalk involves flg22 signaling (Figure 12B). Production of SA is circadian regulated [75], however, activation of SA defense does not affect clock activity (Figure 11 and S8 and [74]). Therefore, SA is an output of the circadian clock but does not serve as an input factor. Since our data showed that CCA1 and LHY act largely independently of SA, we speculate that other circadian clock components may act through SA as an output in defense control.

What would be the advantages for plants to have clock-defense crosstalk? A properly tuned circadian clock enhances growth vigor and confers better survival rate and competitive advantage [11], [12], [13], [14], [15], [16]. Regulation of defense by the circadian clock suggests that timing of effective defense against pathogens is crucial for host fitness in the presence of pathogens. However, defense is an energy-costly process intricately connected with plant growth and development. A feedback regulation of the circadian clock by defense activation could be important for the host to balance growth, development, and defense responses, for instance, to redirect the energy from costly disease resistance to primary metabolism. Consistent with this idea, several phytohormones are potential components of the clock-defense circuitry. For instance, auxin regulates clock activity as an input [74] while auxin production and signaling are affected by the circadian clock and thus are clock output events [73], [76], [77]. Other hormones, such as abscisic acid, brassinosteroids, cytokinins, and gibberellic acid, have been shown to serve as clock inputs [74], [78]. Interestingly, cytokinin affects the phase but not the period of the clock [74], [79], [80]. However whether these hormones are also on the output pathways of the circadian clock remains to be investigated. On the other hand, ethylene and jasmonic acid production and/or signaling are on the output pathways of the circadian clock [29], [75], [81], [82], [83] although ethylene does not serve as a clock input in Arabidopsis [82]. The role of jasmonic acid as a clock input is currently unknown. All these phytohormones have been implicated in defense control besides their critical roles in regulating plant growth and development [84], [85], [86]. Therefore further investigating the roles of these phytohormones in clock-defense crosstalk should shed light on the molecular mechanisms by which plants employ to regulate growth, development, and responses to pathogen invasion. Such information could potentially lead to a better control of plant growth and resistance to devastating pathogens, ultimately enhancing productivity of plants.

## Materials and Methods

### Plant materials

Unless otherwise indicated, all plants used on this paper are in the Columbia-0 (Col-0) background and were grown in growth chambers with a 12 hr light/12 hr dark cycle, light intensity at 200 µmol m^−2^ s^−1^, 60% humidity and 22°C. Single mutants (*acd6-1*, *lhy-20*, and *grp7-1*) and plants overexpressing *CCA1* (*CCA1ox*) or *LHY* (*LHYox*) were described previously [11], [35], [39], [48], [52]. *cca1-1* was originally a Wassilewskija allele but was introgressed into Col-0 via five sequential backcrosses. The mutants *cca1-1lhy-20*, *acd6-1cca1-1*, *acd6-1lhy-20*, and *acd6-1cca1-1lhy-20* were made by genetic crosses and confirmed with PCR markers corresponding to individual mutations (Table S4 and [54]). *CCA1ox* (line #34) and *grp7-1* seeds were from Elaine Tobin and James Alfano, respectively.

### Disease resistance assays

*P. syringae* strains were grown at 28°C with King's B medium (10 g proteose peptone, 1.5 g K_2_HPO_4_, 3.2 ml 1 M MgSO_4_, and 5 g glycerol per liter) containing the appropriate antibiotics for selection. Freshly cultured bacteria were collected, washed once, and resuspended to desired final concentrations in 10 mM MgSO_4_ for infiltration and spray infections or in sterile water for stomatal aperture measurement and bioluminescence analysis. For infiltration infection, the bacterial solution was pressured into the abaxial side of the fifth to seventh leaves of a plant with a 1 ml needleless syringe. For spray infection, the bacterial solution was mixed with Silwet L-77 (Lehle Seeds) at a final concentration of 0.04% and sprayed onto plants until the leaf surface was evenly wet. Bacterial growth and disease symptoms were analyzed as described previously [53]. Log transformed bacterial growth was used in statistical analysis.

*Hyaloperonospora arabidopsidis* (*Hpa*) strains were propagated and prepared as previously described [56], [87]. Seven day-old soil-grown seedlings were sprayed with a spore suspension (5×10^4^ spores/ml in water) containing the virulent strain *Hpa* Emco5 or the avirulent strain *Hpa* Emoy2. Seven days post inoculation, sporangiophores on both sides of cotyledons were counted to determine the level of resistance. *Hpa* infections were conducted as blind experiments where plant genotypes were unknown to the experimenters until the completion of the experiments. All bacterial and *Hpa* infection experiments were repeated at least three times unless otherwise indicated.

### Northern blotting

RNA extraction and northern blotting were performed as described [54]. Radioactive probes were made by polymerase chain reaction (PCR) with a specific antisense primer for a gene fragment in the presence of [^32^P] dCTP. Primers used for making probes were listed in Table S4.

### Stomatal aperture measurement

Stomatal aperture was measured with 25-day-old plants as previously described [1]. Briefly, the fifth to seventh leaves were taken at the indicated times and mounted onto a glass slide at the abaxial side using Telesis 5 silicone adhesive (Premiere Products, Inc., CA). The top layer of the leaf was scratched off with a razor blade. Images of at least three random regions of the bottom layer of the leaf were taken immediately with a camera (Canon Digital Rebel xsi, Japan) connected to an inverted microscope (Olympus Model IMT-2). *P. syringae* treatment was performed at ZT4 when plants had been exposed to light for 4 hr to ensure that most stomata opened. The fifth to seventh leaves of plants were collected and immersed in *PmaDG3* or DC3000 resuspensed in sterile water (10^8^ cfu/ml) or in water as mock treatment. At 1 hpi or 3 hpi, treated leaves were harvested and processed for stomata imaging. At least three leaves per genotype and per time point were taken for stomatal images. The stomatal aperture was determined by the ratio between the width and the length of a stoma, which was measured with the assistance of ImageJ (version 1.45).

### Bioluminescence analysis

Seedlings expressing the reporter gene *LUCIFERASE* (*LUC*) under the control of promoters from *CCA1* or *GRP7* (*At2g21660*; also called *CCR2*) [45], [46] were grown on MS media with 2% sucrose in a 12 hr light/12 hr dark cycle at 22°C for 7–10 days. Seedlings were soaked in *P. syringae* resuspended in sterile water in the presence of 0.04% Silwet L-77, flg22 (1 µM or 10 µM), or benzo(1,2,3)thiadiazole-7-carbothioic acid (BTH; a SA agonist) (10 µM or 300 µM), and transferred to 96-well plates containing 200 µl of MS media and 30 µl of a 2.5 mM D-luciferin solution. Mock treatments were conducted with sterile water with or without 0.04% Silwet L-77. The seedlings were subsequently transferred to LL at 22°C. LUC activity was detected at 1 hr intervals for 7 days with a TopCount luminometer (Perkin Elmer Life Sciences) and analyzed with MetaMorph image software [88]. Flg22 was purchased from GenScript USA Inc. and BTH was a kind gift from Robert Dietrich (Syngenta).

### Cotyledon movement assay

For cotyledon movement, surface sterilized Arabidopsis seeds were grown on MS media with 2% sucrose for 6 days in a 12 hr light/12 hr dark cycle at 22°C and were transferred to 24-well cloning plates, one seedling per well. The seedlings were entrained for two more days in the 12 hr light/12 hr cycle at 22°C and were subsequently released into LL at 22°C. Cotyledon movement was recorded with multiple surveillance cameras every 20 min for 7 days and post-run image analysis was performed as described [88].

### Bioinformatic analysis

Up to 3000 bp promoter sequences of 571 genes [43] were downloaded from Athena (http://www.bioinformatics2.wsu.edu/cgi-bin/Athena/cgi/analysis_select.pl) [89]. These genes were grouped into three sets, selected (337 defense-related gene based on microarray experiments), empirical (127 empirical marker genes for various pathogen responses), and normalization (107 non-defense related genes whose expression levels were relatively stable among experiments with pathogen infection) [43]. Promoters of these genes were analyzed for the enrichment of CBS (AA[AC]AATCT) or EE (AAAATATCT) motifs, using the online tool POBO (http://ekhidna.biocenter.helsinki.fi/poxo/pobo/) [44]. Pseudo-clusters of 100 promoters of up to 3000 bp in length of Arabidopsis genes, which do not contain the coding sequences of the neighboring genes and were sampled randomly from the entire Arabidopsis genome with 1000 bootstrap replications, were analyzed to generate the background as a control for each motif. The number of the CBS or EE motifs in gene clusters was quantified, using a Perl program.

## Supporting Information

Figure S1 **Misexpression of** ***CCA1*** **and** ***LHY*** **disrupts clock activity in LL and LD.** (**A**) Shortening of circadian period in *cca1-1lhy-20* in LL. (**B**) Phase change of *ProCCA1:LUC* in *cca1-1* and *lhy-20* mutants and *CCA1ox* plants in LD. Eight-day-old Col-0, *cca1-1*, *lhy-20*, *cca1-1lhy-20*, and *CCA1ox* seedlings expressing *ProCCA1:LUC* were grown from germination in 12 hr light/12 hr dark cycles at 22°C. Bioluminescence was recorded using a Packard TopCount luminometer in LL (**A**) or in LD (**B**) at 22°C. White boxes indicate the light period, black boxes indicate dark periods, and gray boxes indicate subjective dark periods in LL. Panel (**B**) shows normalized bioluminescence traces shown in Figure 1A.
(TIF)

Figure S2 **Bacterial growth in plants infiltrated with** ***PmaDG3*** **in LL.** (**A**) ZT1 infection. (**B**) ZT13 infection. Plants were grown under the same condition as those used in Figure 2. After infiltration with *PmaDG3* at 1×10^5^ CFU/ml, plants were moved to LL. Letters indicate significant difference among the samples (P<0.05; Student's t-test). These experiments were repeated twice with similar results.
(TIF)

Figure S3 **Stomatal aperture at ZT4.** Leaves of uninfected 25-day-old plants grown in a 12 hr light/12 hr dark cycle at 22°C were taken at ZT4 for the measurement of stomatal aperture. Letters indicate significant difference among the samples (P<0.001; Student's t-test). These experiments were repeated three times with similar results.
(TIF)

Figure S4 **Frequency of motif occurrence on gene promoters.** The number of CBS (**A**) or EE motif (**B**) occurrence per promoter region for selected, empirical, and normalization genes was quantified, using a Perl program.
(TIF)

Figure S5 **Expression of** ***GRP7*** **is CCA1-dependent.** Circadian expression of *GRP7*. Twenty five-day-old Col-0, *cca1-1*, and *CCA1ox* plants grown in a chamber with a 12 hr light/12 hr dark cycle and 22°C were transferred to LL at 22°C. Starting from ZT1, plants were harvested at every 4 hr interval for 48 hr for RNA extraction followed by northern blotting. White boxes indicate subjective light periods and gray boxes indicate subjective dark periods in LL. *GRP7* transcripts were shown on the top three panels. 18S rRNA from each genotype at different time points, shown on the bottom three panels, was used as a loading control.
(TIF)

Figure S6 **CCA1 and LHY functions are largely SA-independent.** (**A**) SA quantification. Total SA was extracted from plants and analyzed by HPLC. Data represent the average of SA levels (n = 3) ± standard deviation. (**B**) Picture of 20- and 30-day-old L*er* and *LHYox* plants. The same batch of plants were used in Figure 4C and 4D for SA and cell death analyses.
(TIF)

Figure S7 **Defense activation by** ***P. syringae*** **infection shortens the period of the** ***GRP7:LUC*** **reporter.** Eight-day-old Col-0 seedlings expressing the *ProGRP7:LUC* reporter were grown from germination in 12 hr light/12 hr dark cycle at 22°C. Then the seedlings were infected with *PmaDG3* or *PmaDG6* at OD = 0.1 (1×10^8^ CFU/ml) or OD = 0.01 (1×10^7^ CFU/ml) and transferred to 96-well plates containing 200 µl of MS media and 30 µl of a 2.5 mM D-luciferin solution. Luciferase activity was recorded with a Packard TopCount luminometer in LL at 22°C. (**A**) Mean circadian traces for *ProGRP7:LUC* activity. White bars indicate subjective day and gray bars indicate subjective night. (**B**) Mean circadian period of the *ProGRP7:LUC* reporter. SEM (n = 12–24) was used for (**A**) and (**B**). These experiments were repeated twice with similar results.
(TIF)

Figure S8 **Cotyledon movement assay with** ***acd6-1*** **.** (**A**) Mean circadian period of cotyledon movement of *acd6-1*. (**B**) Summary of period, phase, RAE, and amplitude.
(TIF)

Table S1 **Suppression of stomatal aperture in the presence of** ***P. syringae*** **.**
(DOCX)

Table S2 **Fold change of cluster means of motif enrichment.**
(DOCX)

Table S3 **Defense activation by** ***P. syringae*** **infection or flg22 treatment shortens the clock period.**
(DOCX)

Table S4 **Primers used in this paper.**
(DOCX)
