# Supplementary figures and images for: Crosstalk between the Circadian Clock and Innate Immunity in Arabidopsis
Source: PLoS Pathog. 2013 Jun 6;9(6):e1003370. doi: 10.1371/journal.ppat.1003370 (PMC3675028; doi:10.1371/journal.ppat.1003370)

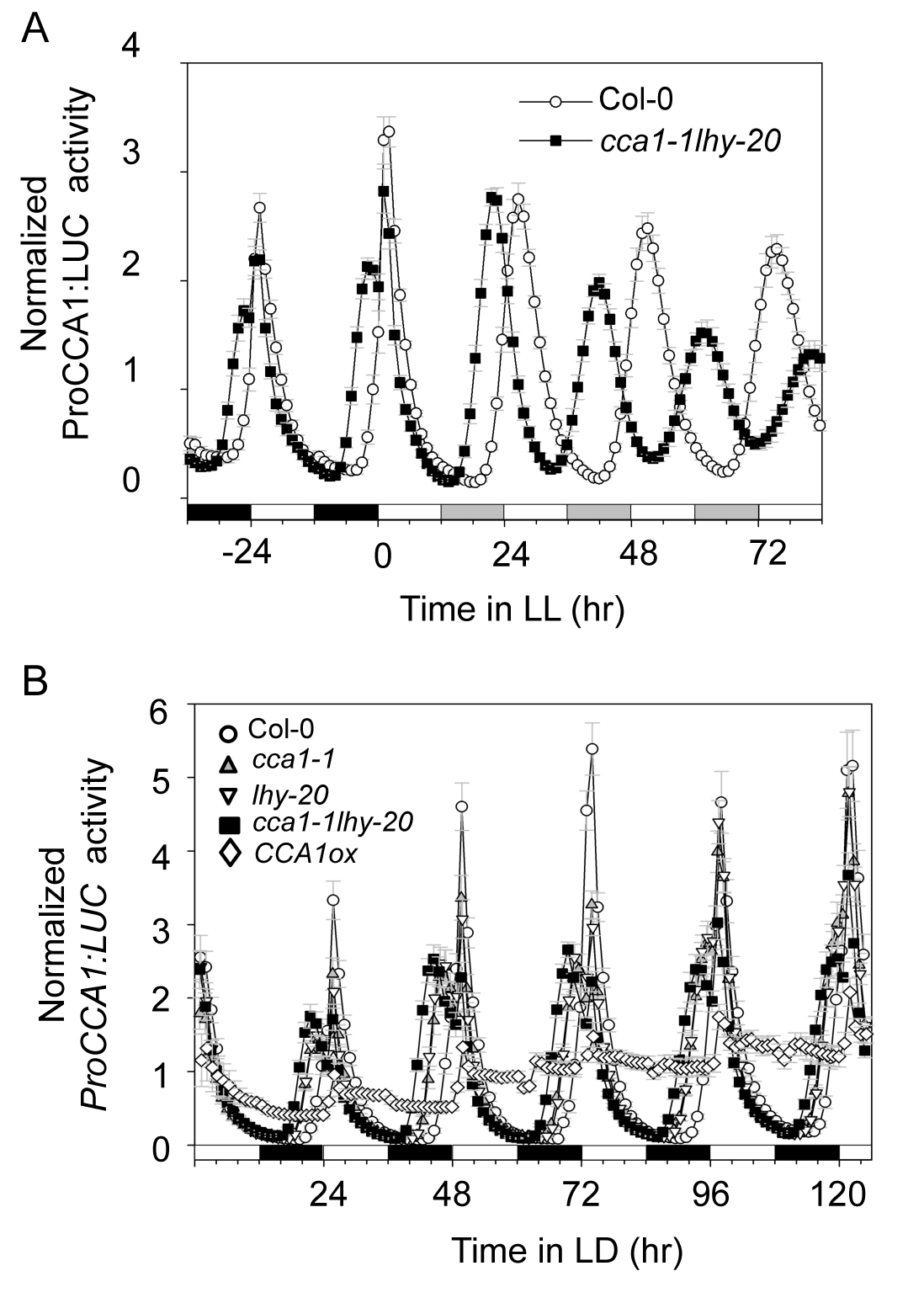

Supplement: Figure S1 — Misexpression of CCA1 and LHY disrupts clock activity in LL and LD. (A) Shortening of circadian period in cca1-1lhy-20 in LL. (B) Phase change of ProCCA1:LUC in cca1-1 and lhy-20 mutants and CCA1ox plants in LD. Eight-day-old Col-0, cca1-1, lhy-20, cca1-1lhy-20, and CCA1ox seedlings expressing ProCCA1:LUC were grown from germination in 12 hr light/12 hr dark cycles at 22°C. Bioluminescence was recorded using a Packard TopCount luminometer in LL (A) or in LD (B) at 22°C. White boxes indicate the light period, black boxes indicate dark periods, and gray boxes indicate subjective dark periods in LL. Panel (B) shows normalized bioluminescence traces shown in Figure 1A. (TIF) [file ppat.1003370.s001.tif]

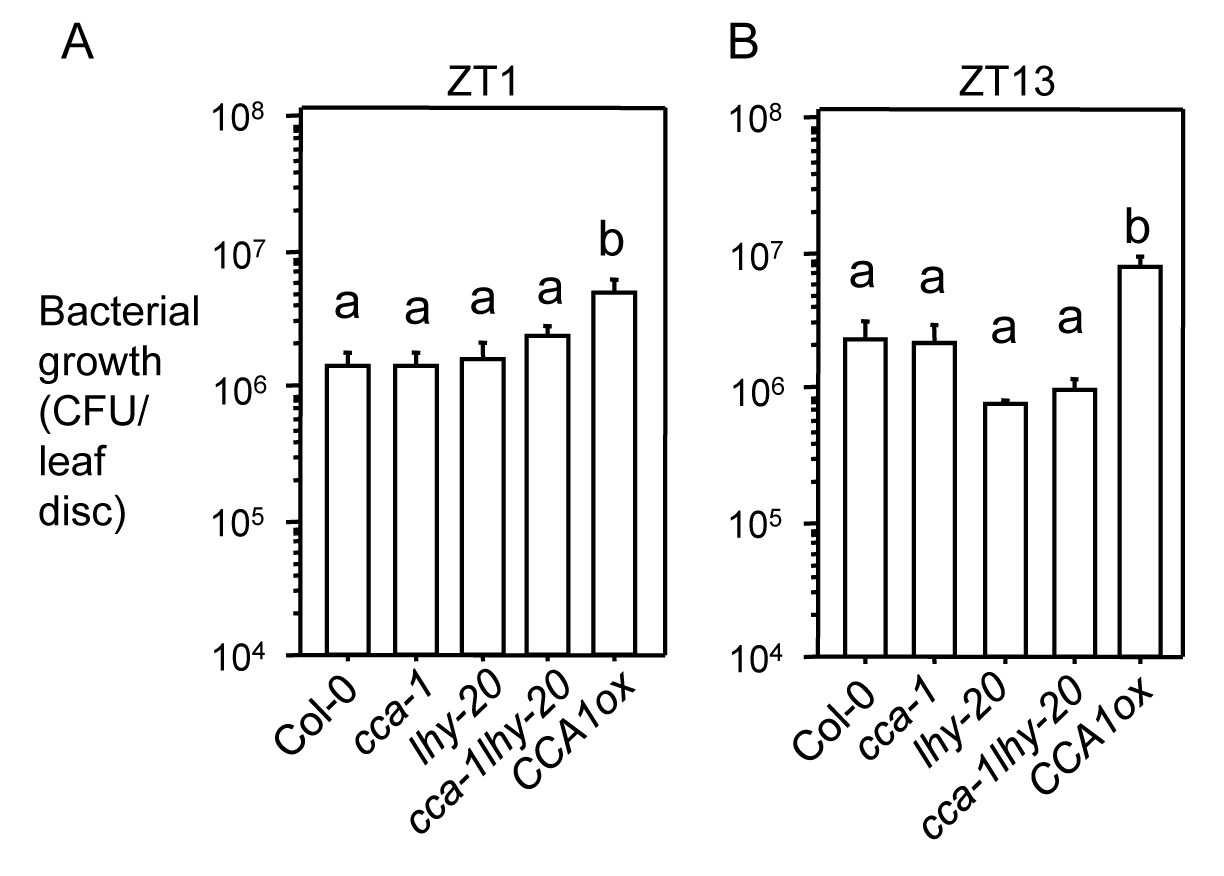

Supplement: Figure S2 — Bacterial growth in plants infiltrated with PmaDG3 in LL. (A) ZT1 infection. (B) ZT13 infection. Plants were grown under the same condition as those used in Figure 2. After infiltration with PmaDG3 at 1×10^5^ CFU/ml, plants were moved to LL. Letters indicate significant difference among the samples (P<0.05; Student's t-test). These experiments were repeated twice with similar results. (TIF) [file ppat.1003370.s002.tif]

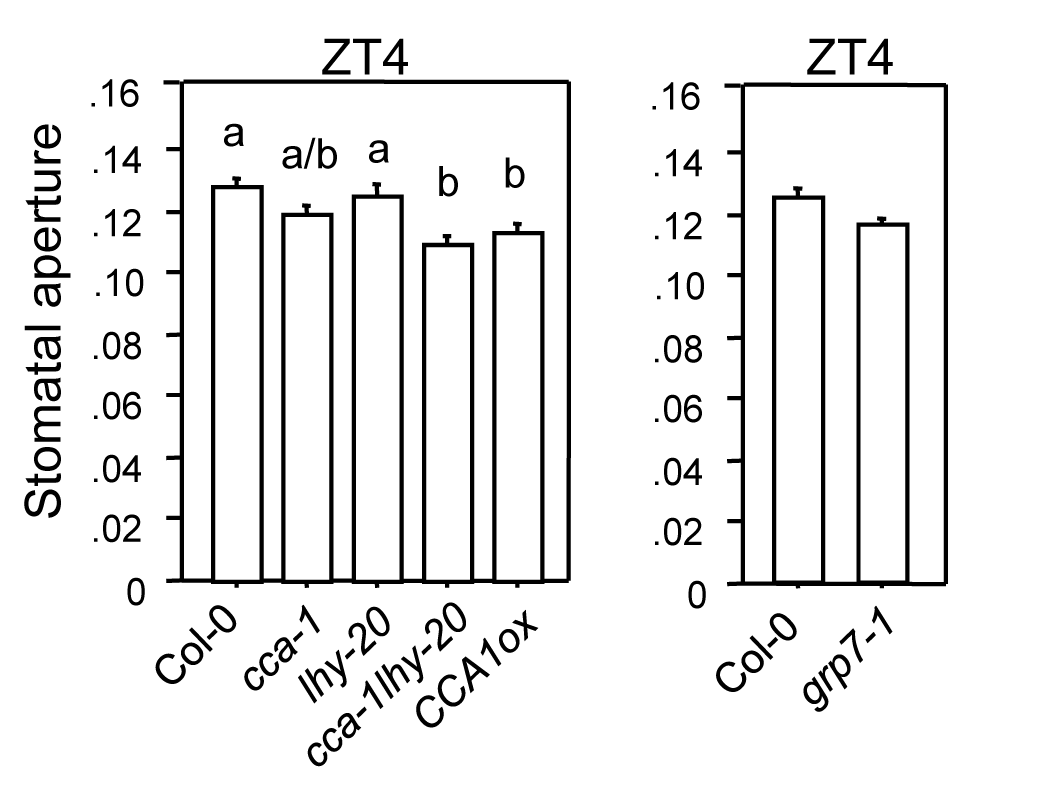

Supplement: Figure S3 — Stomatal aperture at ZT4. Leaves of uninfected 25-day-old plants grown in a 12 hr light/12 hr dark cycle at 22°C were taken at ZT4 for the measurement of stomatal aperture. Letters indicate significant difference among the samples (P<0.001; Student's t-test). These experiments were repeated three times with similar results. (TIF) [file ppat.1003370.s003.tif]

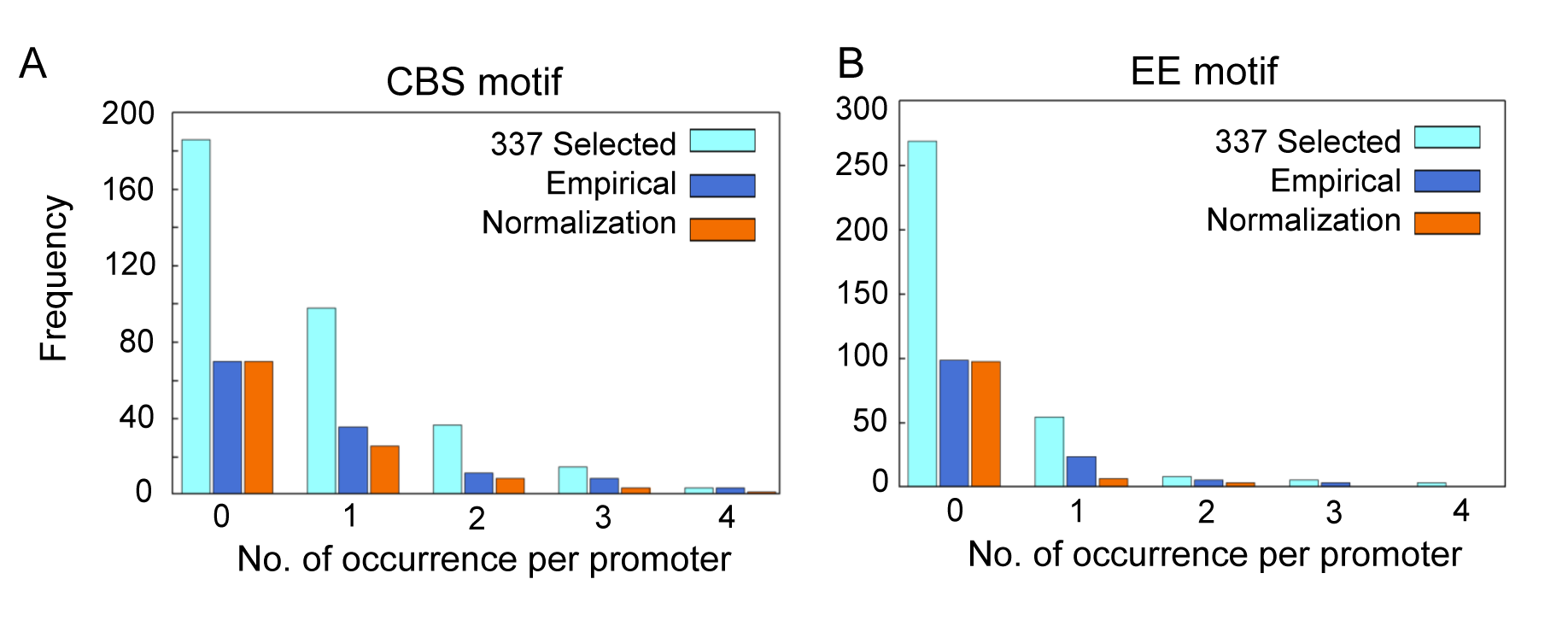

Supplement: Figure S4 — Frequency of motif occurrence on gene promoters. The number of CBS (A) or EE motif (B) occurrence per promoter region for selected, empirical, and normalization genes was quantified, using a Perl program. (TIF) [file ppat.1003370.s004.tif]

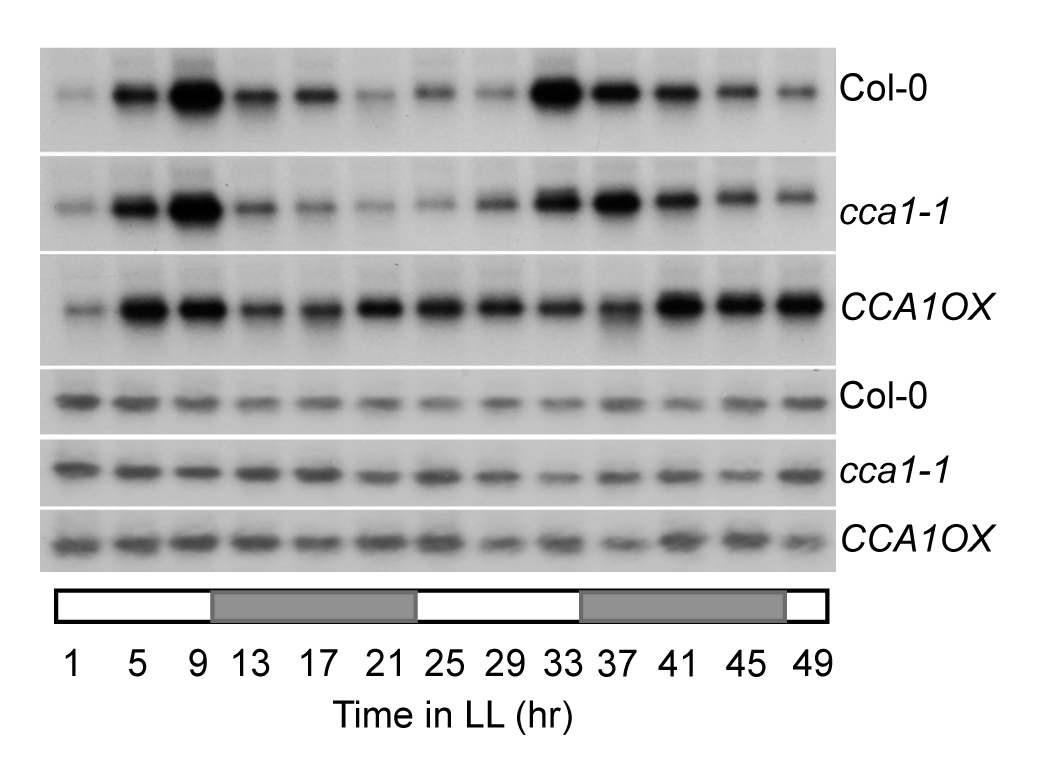

Supplement: Figure S5 — Expression of GRP7 is CCA1-dependent. Circadian expression of GRP7. Twenty five-day-old Col-0, cca1-1, and CCA1ox plants grown in a chamber with a 12 hr light/12 hr dark cycle and 22°C were transferred to LL at 22°C. Starting from ZT1, plants were harvested at every 4 hr interval for 48 hr for RNA extraction followed by northern blotting. White boxes indicate subjective light periods and gray boxes indicate subjective dark periods in LL. GRP7 transcripts were shown on the top three panels. 18S rRNA from each genotype at different time points, shown on the bottom three panels, was used as a loading control. (TIF) [file ppat.1003370.s005.tif]

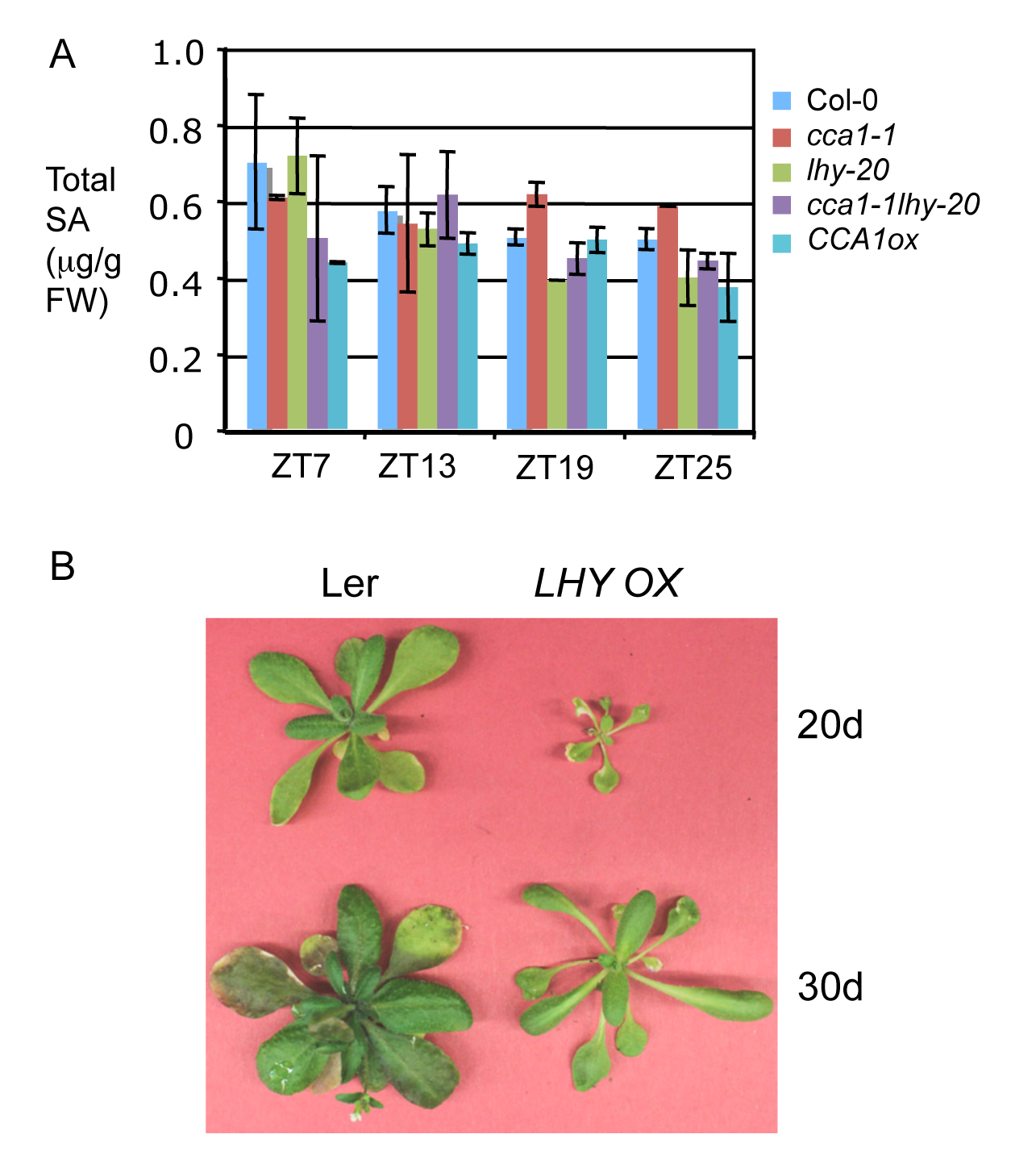

Supplement: Figure S6 — CCA1 and LHY functions are largely SA-independent. (A) SA quantification. Total SA was extracted from plants and analyzed by HPLC. Data represent the average of SA levels (n = 3) ± standard deviation. (B) Picture of 20- and 30-day-old Ler and LHYox plants. The same batch of plants were used in Figure 4C and 4D for SA and cell death analyses. (TIF) [file ppat.1003370.s006.tif]

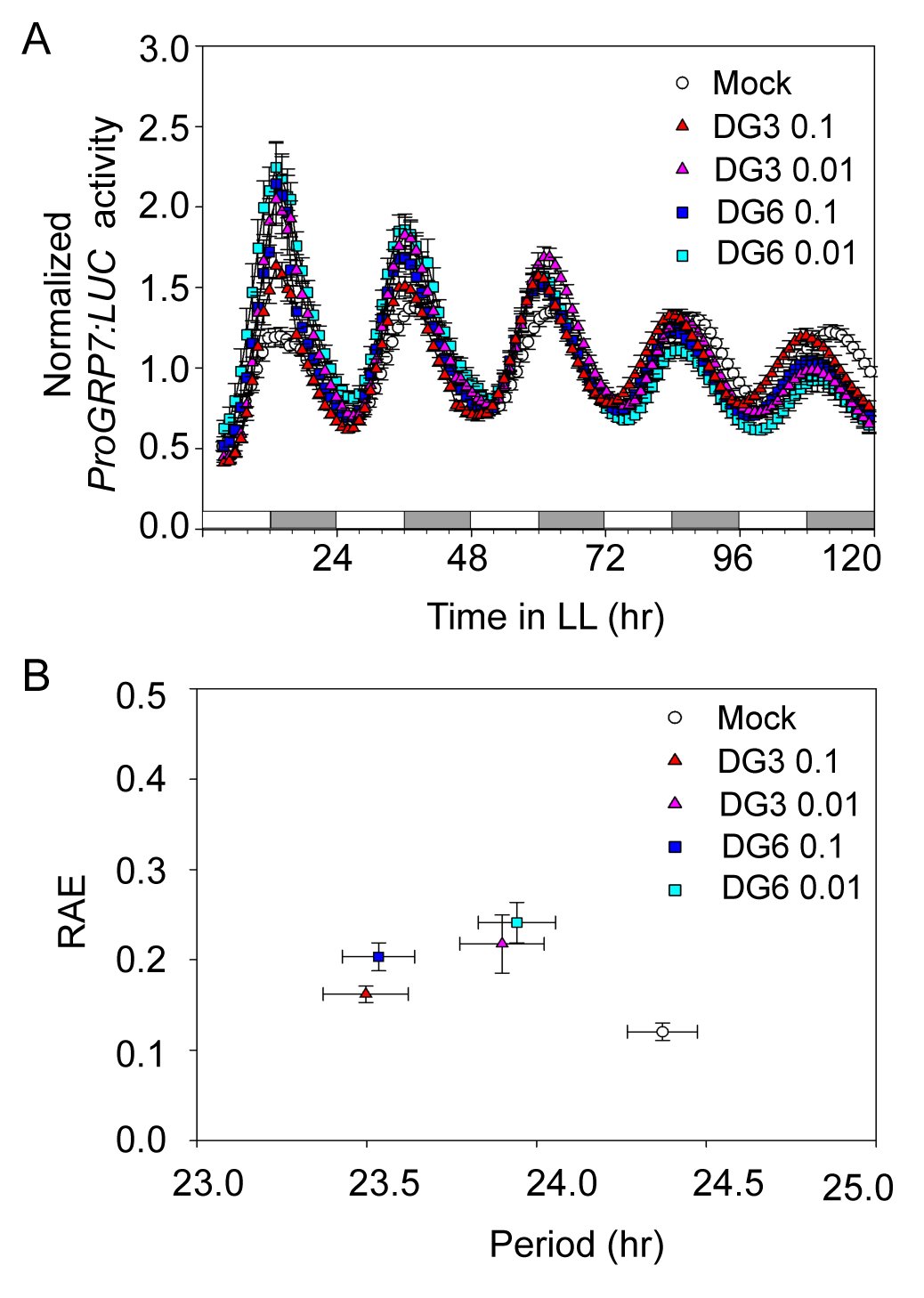

Supplement: Figure S7 — Defense activation by P. syringae infection shortens the period of the GRP7:LUC reporter. Eight-day-old Col-0 seedlings expressing the ProGRP7:LUC reporter were grown from germination in 12 hr light/12 hr dark cycle at 22°C. Then the seedlings were infected with PmaDG3 or PmaDG6 at OD = 0.1 (1×10^8^ CFU/ml) or OD = 0.01 (1×10^7^ CFU/ml) and transferred to 96-well plates containing 200 µl of MS media and 30 µl of a 2.5 mM D-luciferin solution. Luciferase activity was recorded with a Packard TopCount luminometer in LL at 22°C. (A) Mean circadian traces for ProGRP7:LUC activity. White bars indicate subjective day and gray bars indicate subjective night. (B) Mean circadian period of the ProGRP7:LUC reporter. SEM (n = 12–24) was used for (A) and (B). These experiments were repeated twice with similar results. (TIF) [file ppat.1003370.s007.tif]

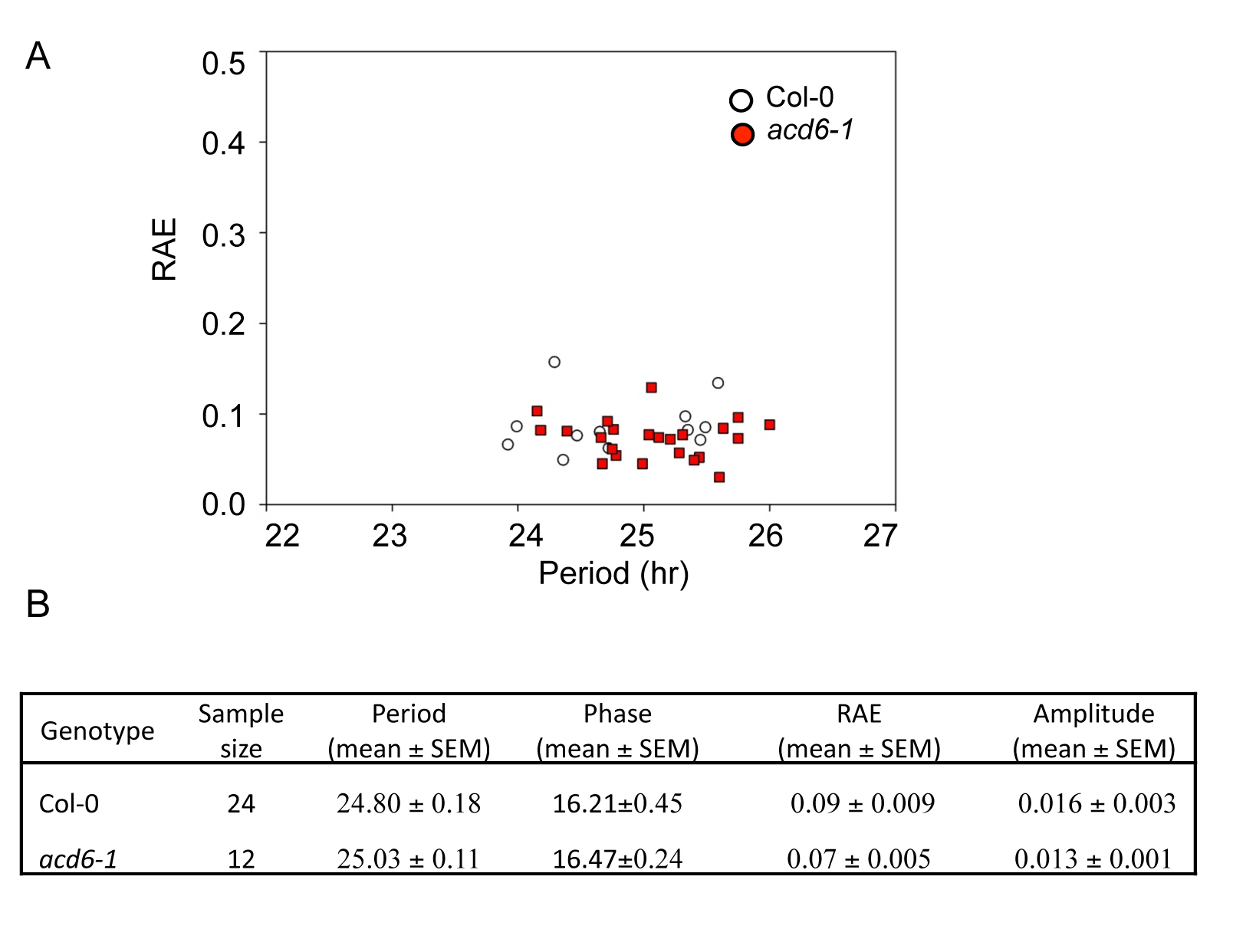

Supplement: Figure S8 — Cotyledon movement assay with acd6-1 . (A) Mean circadian period of cotyledon movement of acd6-1. (B) Summary of period, phase, RAE, and amplitude. (TIF) [file ppat.1003370.s008.tif]
