# Supplementary material for: Crosstalk between the Circadian Clock and Innate Immunity in Arabidopsis
Source: PLoS Pathog. 2013 Jun 6;9(6):e1003370. doi: 10.1371/journal.ppat.1003370 (PMC3675028; doi:10.1371/journal.ppat.1003370)
Supplement: Table S1 — Suppression of stomatal aperture in the presence of P. syringae . (DOCX) [file ppat.1003370.s009.docx]

**Table S1.** **Suppression of stomatal aperture in the presence of *P. syringae*.** Suppression of stomatal opening in each genotype at 1 hpi or 3 hpi was calculated based on the data shown in Figure 5C, 5D, and 8C. Suppression = (Stomatal aperture (Mock) - Stomatal aperture (PmaDG3))*100/ Stomatal aperture (Mock). PmaDG3 was used to infect Col-0, *cca1-1*, *lhy-20*, *cca1-1lhy-20*, and *CCA1ox* while DC3000 was used to infect L*er* and *LHYox*.

|  | Suppression of stomatal aperture (%) | |
| --- | --- | --- |
| Genotypes | 1 hr infection | 3 hr infection |
| Col-0 | 48.1 | 7.4 |
| *cca1-1* | 28.6 | 6.0 |
| *lhy-20* | 19.0 | 9.0 |
| *cca1-1lhy-20* | 7.0 | -1.5 |
| *CCA1ox* | 5.1 | 2.1 |
| *grp7-1* | 14.2 | 7.1 |
| L*er* | 51.6 | -4.8 |
| *LHYox* | 16.9 | 16.8 |
