# Supplementary material for: Crosstalk between the Circadian Clock and Innate Immunity in Arabidopsis
Source: PLoS Pathog. 2013 Jun 6;9(6):e1003370. doi: 10.1371/journal.ppat.1003370 (PMC3675028; doi:10.1371/journal.ppat.1003370)
Supplement: Table S2 — Fold change of cluster means of motif enrichment. (DOCX) [file ppat.1003370.s010.docx]

**Table S2.** **Fold change of cluster means of motif enrichment.** A total of 571 promoters from genes in three categories, selected (337 defense-related gene based on microarray experiments), empirical (127 empirical marker genes for various pathogen responses), and normalization (107 non-defense related genes) was analyzed for the enrichment of CBS or EE motifs, using the online tool POBO (<http://ekhidna.biocenter.helsinki.fi/poxo/pobo/>) (Kankainen and Holm, 2004). The mean of motif enrichment for each cluster was used to derive the fold change between different gene categories. Fold over Background = (mean of selected, empirical, or normalization – mean of background)*100/mean of background. Fold over normalization = (mean of selected or empirical – mean of normalization)*100/mean of normalization.

| Genotypes | CBS Mean | Fold over Background (%) | Fold over Normalization (%) | EE Mean | Fold over Background (%) | Fold over Normalization (%) |
| --- | --- | --- | --- | --- | --- | --- |
| Background | 61.5 |  |  | 14.4 |  |  |
| 337 selected | 65.8 | 6.9 | 40.7 | 25.7 | 78.4 | 203.8 |
| Empirical | 68.4 | 11.3 | 46.4 | 24.6 | 70.7 | 190.6 |
| Normalization | 46.7 | -24.0 | 0.0 | 8.5 | -41.3 | 0.0 |

**Reference**

**Kankainen, M., and Holm, L.** (2004). POBO, transcription factor binding site verification with bootstrapping. Nucleic Acids Res **32**: W222-229.
