# Supplementary material for: Crosstalk between the Circadian Clock and Innate Immunity in Arabidopsis
Source: PLoS Pathog. 2013 Jun 6;9(6):e1003370. doi: 10.1371/journal.ppat.1003370 (PMC3675028; doi:10.1371/journal.ppat.1003370)
Supplement: Table S3 — Defense activation by P. syringae infection or flg22 treatment shortens the clock period. (DOCX) [file ppat.1003370.s011.docx]

**Table S3.** **Defense activation by *P. syringae* infection or flg22 treatment shortens the clock period.** Eight-day-old Col-0 seedlings expressing the *ProCCA1:LUC* reporter or the *ProGRP7:LUC* reporter were grown from germination in a chamber with a 12 hr light/12 hr dark cycle at 22°C. Then the seedlings were infected with *PmaDG3* or *PmaDG6* at OD=0.1 (1x10^8^ CFU/ml) or OD=0.01 (1x10^7^ CFU/ml)*.* Alternatively, the seedlings were treated with flg22 (1 μM or 10 μM) or BTH (10 μM or 300 μM) and transferred to 96-well plates containing 200 µl of MS media and 30 µl of a 2.5 mM D-luciferin solution in LL at 22°C. Luciferase activity was recorded with a Packard TopCount luminometer. Mean circadian period of each reporter ± SEM is shown. Statistical analysis was performed by One-way ANOVA. Asterisks indicate significant difference between the treated samples and the mock control (P<0.05).

| Treatment | *ProCCA1:LUC*  Period ± SEM (n) | *ProGRP7:LUC*  Period ± SEM (n) |
| --- | --- | --- |
| Mock | 24.37±0.06 (24) | 24.37±0.10 (23) |
| *Pma*DG3 0.01 | 24.18±0.10 (23) | 23.90±0.12* (24) |
| *Pma*DG3 0.1 | 23.89±0.16* (21) | 23.50±0.13* (24) |
| *Pma*DG6 0.01 | 24.24±0.11 (24) | 23.94±0.11 (24) |
| *Pma*DG6 0.1 | 23.78±0.10* (24) | 23.54±0.11* (24) |
| Mock | 24.68±0.07 (20) |  |
| BTH 10μM | 24.43±0.12 (12) |  |
| BTH 300μM | 24.43±0.08 (11) |  |
| flg22 1μM | 24.23±0.10* (11) |  |
| flg22 10μM | 24.12±0.09* (11) |  |
