# Supplementary material for: Crosstalk between the Circadian Clock and Innate Immunity in Arabidopsis
Source: PLoS Pathog. 2013 Jun 6;9(6):e1003370. doi: 10.1371/journal.ppat.1003370 (PMC3675028; doi:10.1371/journal.ppat.1003370)
Supplement: Table S4 — Primers used in this paper. (DOCX) [file ppat.1003370.s012.docx]

**Table S4. Primers used in this paper.**

|  | **Primer sequence (5'>3')** | **Note** |
| --- | --- | --- |
| **Primer sets used for genotyping** | |  |
| *cca1-1* | AGGCTTTATGGTAGAGCATGGCA  AGCTTCAGCCTCTTTCTCTACCTGA  AACGTCCGCAATGTGTTATTAAGTTGTC | T-DNA insertion |
| *lhy-20* | ACATAGAAATTCCGCCTCCTCGT  AGATTTGCGTGCCCGTGAGT  CATTTTATAATAACGCTGCGGACATCTAC | T-DNA insertion |
| **Primer sets used to make probes for northern blotting** | | |
| *GRP7* | GTTACTCCGGTGGAGGTGGTAG  AAATCTAAAGCAGAACCAGAACAAA | |
| *18S* | GATAACTCGACGGATCGCATGG  CCTAAACGGCCATAGTCCCTC | |
